# A spectrum of tectonic processes at coronae on Venus revealed by gravity and topography

**DOI:** 10.1126/sciadv.adt5932

**Published:** 2025-05-14

**Authors:** Gael Cascioli, Anna J. P. Gülcher, Erwan Mazarico, Suzanne E. Smrekar

**Affiliations:** ^1^ NASA Goddard Space Flight Center, Greenbelt, MD, USA.; ^2^ Center for Space Science and Technology, University of Maryland Baltimore County, Baltimore, MD, USA.; ^3^ Center for Space and Habitability, University of Bern, Bern, Switzerland.; ^4^ Division of Geological and Planetary Sciences, California Institute of Technology, Pasadena, CA, USA.; ^5^ Jet Propulsion Laboratory, California Institute of Technology, Pasadena, CA, USA.

## Abstract

Coronae on Venus are key to understanding the planet’s geodynamics. Their formation is often linked to plume-lithosphere interactions, with some coronae showing signs of plate boundary-like processes such as subduction. However, the low resolution of Venus gravity data limits detailed analysis of these features. Using 3D geodynamic models, we predict gravity signals under various plume-induced corona formation scenarios. Comparing these predictions to observations, we show that combining topography and gravity data is more effective for understanding dynamic processes than using topography alone. Of the 75 resolved coronae, gravity indicates buoyant mantle material beneath 52. We predict a range of plume-lithosphere interactions and activity stages across these coronae. Moreover, we find that the limited resolution of the Magellan gravity field can obscure gravity signatures otherwise indicative of plume activity. The upcoming VERITAS mission will greatly improve gravity resolution, which will resolve 427 coronae, enhancing our understanding of Venus’ lithospheric structure and geodynamics.

## Teaser

Gravity and topography data reveal diverse mantle activity styles beneath Venus’ coronae and potentially hidden signals.

## INTRODUCTION

Understanding the interior processes shaping Venus’ surface is a fundamental goal for planetary sciences. Venus, Earth’s “twin planet,” presents clear contrasts in terms of surface conditions, atmospheric chemistry, and tectonic state. Today, Venus does not feature Earth-like plate tectonics. Instead, its surface deformation is likely driven by mantle convection and plume-lithosphere interactions [e.g., (*1*)]. Recent studies indicate several geologically active sites [e.g., (*2*–*4*)], suggesting more Earth-like activity than originally assumed. Despite this changing perspective, much remains unknown about Venus’ current tectonic state, which several new missions are set to investigate.

**Fig. 1.**
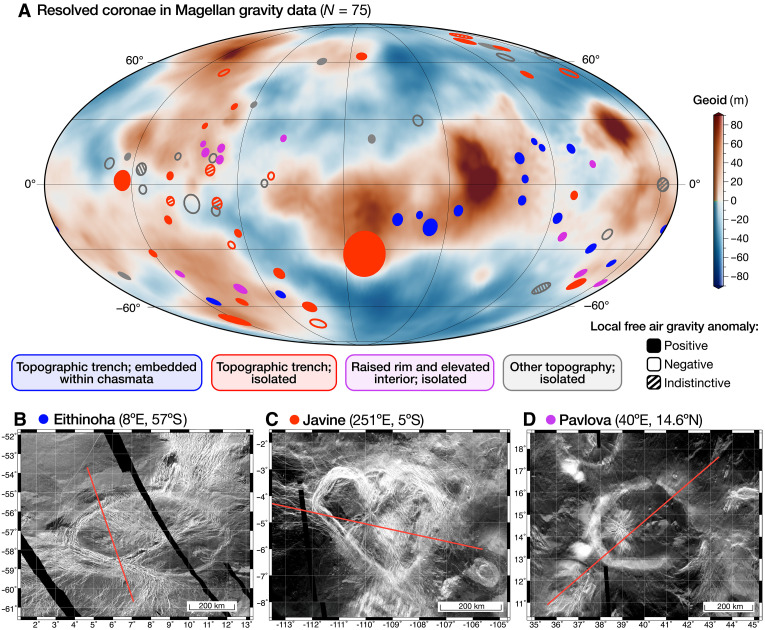
Global distribution and classification of coronae resolved in Magellan data and examples of active coronae. (**A**) Map in Mollweide projection centered at 130°E displaying the geoid derived from the Magellan MGNP180U gravity field solution (*25*) and the 75 coronae considered broadly or finely resolved in the Magellan gravity data. These coronae have topographic radii equal to or larger than the local Magellan gravity field resolution, ensuring at least one independent gravity data point within their radius (see Methods). Coronae are plotted based on their central coordinates and average radii [from (*5*)]. The filling and outlining of the circles indicate the type of free-air gravity anomaly associated with each corona; colors represent their topographic classification. We propose the coronae with positive free-air gravity anomalies (filled circles) to be active, i.e., sites of ongoing plume-lithosphere interactions of different styles based on our findings. Crustal recycling processes are inferred for active coronae with (partial) topographic trenches (dark blue and red solid fills); “embedded/underplated plume” scenarios are inferred for those with a raised rim and elevated interior (pink solid fill) (see Results and Discussion for more details). (**B** to **D**) Three coronae that exemplify the three major active coronae groups, with a map of Magellan left-cycle SAR imagery (*60*) in cylindrical equidistant projection. One of the profiles used for fitting to geodynamic models (Fig. 6) is shown in red.

We propose that a spectrum of active plume-lithosphere interaction processes occur at so-called “coronae,” quasi-circular volcano-tectonic features characterized by a (partial) annulus of concentric fractures (Fig. 1, B to D). The 740 cataloged coronae (*5*) are widely distributed across Venus’ surface, with varying dimensions (diameters of 60 to 2500 km), morphologies, and geological settings (*5*–*7*). Corona formation scenarios often involve lithospheric responses above buoyant or transient mantle plumes (*8*–*16*) or gravitational instabilities and lithospheric downwellings (*17*, *18*). The diversity of coronae suggests that they form via various mechanisms, making corona tectonics key to understanding Venus’ global tectonic regime and resurfacing history (*1*).

Modeling studies aimed at matching simulated corona morphologies with observations (*11*, *14*–*21*) typically reproduce some key corona features such as topography and/or gravity signatures and point to a progression of topographic profiles over time. Among these, three-dimensional (3D) numerical simulations of plume-induced corona formation by (*15*) defined four geodynamic end-member scenarios: (i) lithospheric dripping, (ii) subduction, (iii) embedded plume, and (iv) underplated plume. Narrow topographic trenches were captured by models with down-going lithosphere and crustal recycling at the plume margin [lithospheric dripping or subduction in (*15*)]. These narrow topographic trenches are displayed by ∼20% of all coronae on Venus, typically in large ones (diameters ≥300 km) (*5*). Trenches at several large corona margins have been suggested as sites of active plume-induced subduction (*13*, *15*, *22*). However, alternative sequences of topographic development have been proposed, in which trenches are placed in the final, inactive stages of corona evolution, as suggested by conceptual models (*10*).

Establishing direct causal relationships between observed topography and dynamic processes is challenging, especially with inadequate data resolution and the nonunique link between topography and underlying density structures and dynamics. However, gravity data offer invaluable insights into subsurface and interior density variations. An analysis of peak free-air gravity anomalies over 428 coronae identified 125 as uncompensated, suggesting ongoing dynamic processes, although specific mechanisms were not explored (*23*). Forward 2D modeling studies showed that negative and positive free-air anomalies correlate with down- and upwellings, respectively, with amplitudes influenced by crustal and lithospheric properties (*17*). Moreover, predicted Bouguer anomalies highlighted subsurface density variations due to compositional or thermal anomalies (*14*, *18*). Comparison of these predicted gravity signatures with actual observations, however, is limited, likely due to the coarse resolution of Venus global gravity data (Magellan mission, 1989–1994). Moreover, recent geodynamic models incorporating advanced complexities such as non-Newtonian rheologies, melting, and magmatism in 3D [e.g., (*15*, *20*)] or two-phase flow laws (*16*) have not yet been applied in forward gravity modeling, leaving these scenarios for corona formation unexplored in terms of gravity.

Here, we combine specific geodynamic models of plume-induced formation of large coronae on Venus (*15*), forward gravity modeling, and datasets from the Magellan mission and the upcoming Venus Emissivity, Radio Science, InSAR, Topography and Spectroscopy (VERITAS) mission to reveal current and forthcoming insights into ongoing tectonic processes at coronae on Venus.

Among the 75 coronae resolved in Magellan gravity data (Fig. 1A), gravity indicates the presence of buoyant mantle material beneath 52, which we propose to be “active” (i.e., ongoing plume-lithosphere interactions characterized by the presence of thermal anomalies and subsurface material flow; see Methods). Based on quantitative comparisons between models and observations, we suggest a spectrum of plume-lithosphere interactions and activity stages at these coronae (Fig. 1A), going substantially beyond previous coronae studies. We also demonstrate that the limited resolution of Magellan gravity data can obscure positive gravity anomalies at some coronae, potentially hindering their interpretation. Last, with the anticipated improved resolution of the VERITAS gravity dataset, we predict that an impressive 427 coronae will be resolved and identify sites of interest for the upcoming missions.

## RESULTS

### Geodynamic evolution scenarios and forward gravity predictions

**Fig. 2.**
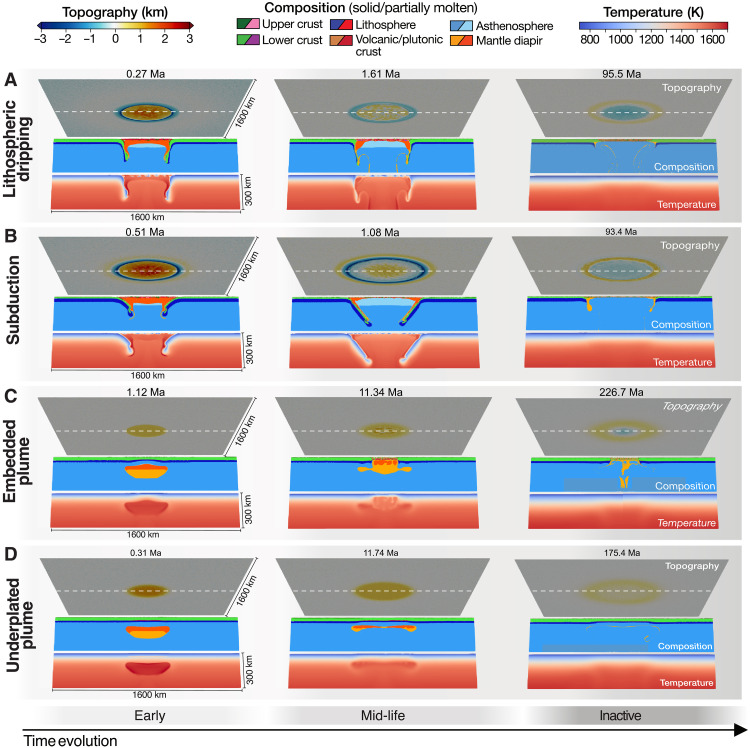
Evolution of four geodynamic end-member models that form coronae at the surface. The end-member geodynamic styles, defined by (*15*), are: (**A**) lithospheric dripping, (**B**) short-lived subduction, (**C**) embedded plume, and (**D**) underplated plume (see Results). Each timestep includes a map of surface topography (top) and a cross section of composition (middle) and temperature (bottom) taken in the center of the model (dashed line) (see legend). In contrast to active geodynamic stages (ongoing plume-lithosphere interactions), inactive stages (right panels) are characterized by insufficient thermal anomalies and material flow beneath the corona surface (see thresholds in Methods). Ma, model time in million years.

Here, we run the representative models of the four end-member styles of plume-lithosphere interactions defined in (*15*) (lithospheric dripping, subduction, embedded plume, and underplated plume) for longer durations and analyze them to explore the geodynamics and gravity signatures of active-to-inactive stages (see Methods). In all models, the buoyant mantle plume first rises closer toward the surface. When the plume is buoyant enough to break the lithosphere, it impinges upon the crust and pushes crustal and lithospheric material outwards and downwards. The displaced lithosphere at the plume margin either causes (i) repeated lithospheric dripping for a relatively weak lithosphere (Fig. 2A and fig. S1A) or a (ii) short-lived, retreating radial subduction zone for a stronger lithosphere (Fig. 2B and fig. S1B).

For slightly less buoyant plumes and/or stronger lithosphere, the plume may become (iii) terminally embedded underneath the lithosphere or crust without crustal material recycling (Fig. 2C and fig. S1C). Last, if the plume is not buoyant enough or the lithosphere is too strong, then it (iv) underplates beneath the lithosphere, causing a topographic signature that remains elevated (Fig. 2D and fig. S1D).

**Fig. 3.**
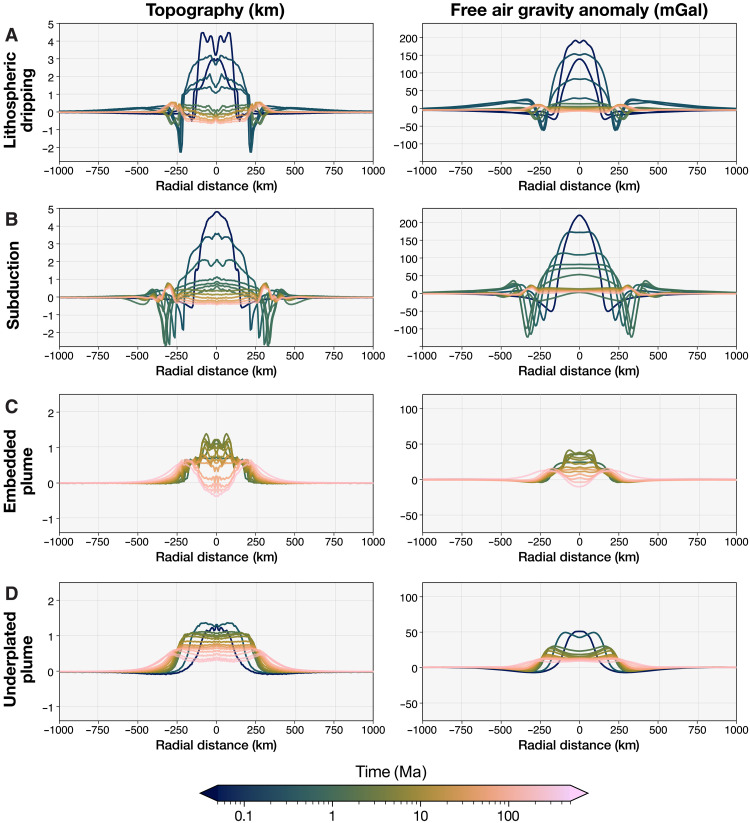
Temporal evolution of radial topography and gravity of geodynamic models. The panels show the evolution of the radially-averaged profiles in topography (left) and free-air gravity anomaly (right) for the four geodynamic end-member cases: (**A**) lithospheric dripping, (**B**) short-lived subduction, (**C**) embedded plume, and (**D**) underplated plume (see Results). The temporal evolution of the radial Bouguer gravity anomaly for the same cases can be found in fig. S4. The color bar uses a logarithmic scale to represent time, effectively capturing the model’s rapid initial evolution, related to fast changes in topography and gravity due to tectonic processes, followed by slower dynamics, where changes in topography and gravity are primarily driven by lithospheric cooling. In addition, note the distinct vertical axes for the crustal recycling scenarios [dripping/subduction, shown in (A) and (B)] compared to the embedded/underplated plume cases (C and D).

For selected snapshots through time, we calculate the free-air and Bouguer gravity anomalies using the modeled topography and 3D density fields (Fig. 2), which depend on composition, temperature, pressure, and melt content (see Methods). All four geodynamic cases exhibit variations in free-air gravity anomaly morphology through time (Fig. 3 and fig. S2), closely following the topographic evolution. Initially, the rising buoyant mantle plume creates a central high free-air gravity anomaly surrounded by a ring of negative anomaly. At the peak of tectonic activity, the crustal recycling models (Fig. 3, A and B. and fig. S2, A and B) display an outer free-air gravity high associated with the topographic rise, a narrow and deep circular gravity low corresponding to the topographic trench, and a high central anomaly related to the elevated central topography caused by the buoyant plume. As these models evolve, the ring of negative gravity anomalies (trench) transitions to a ring of positive anomalies (rim), reflecting the topographic inversion seen at inactive stages (Fig. 2). Conversely, models with embedded and underplated plumes show only a broad ring of minor negative free-air gravity anomaly surrounding a central high anomaly during active stages (Fig. 3, C and D, and fig. S2, C and D). The Bouguer anomalies in the geodynamic models differ from the free-air anomalies, predominantly remaining negative over time (figs. S3 and S4), indicating substantial compensation.

### Comparing predictions to observations at different resolutions

**Fig. 4.**
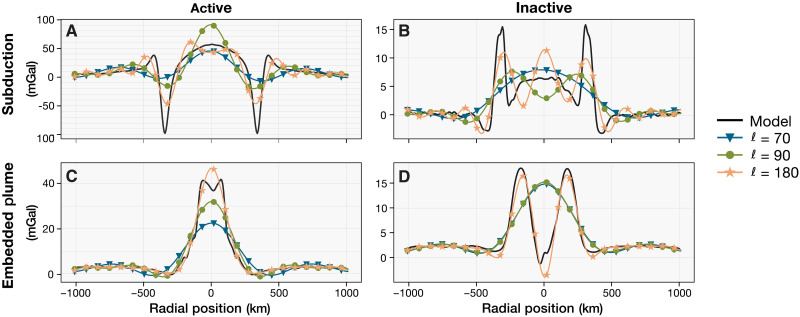
Effect of spatial resolution on synthetic gravity data: Comparing Magellan and VERITAS. Comparison of the free-air gravity anomaly of selected geodynamic model snapshots at the full resolution (6.8-km grid), typical Magellan resolution (spherical harmonics degrees 70 to 90), and expected baseline VERITAS resolution (ℓ>179 over >90% of the planet). The specific models and timesteps shown are: subduction (**A**) *t* = 1.1 Ma and (**B**) *t* = 131.5 Ma; embedded plume (**C**) *t* = 4.2 Ma and (**D**) *t* = 135.4 Ma. The Magellan gravity dataset can only capture the large-scale features of coronae gravity signatures, such as peak amplitudes, while the future VERITAS dataset is expected to offer much more detail. The modeled coronae structures discussed represent some of the largest observed on Venus, with radial extents reaching from 400 up to 800 km. Because most coronae on Venus have smaller diameters, the impact of resolution differences will be even more pronounced. Note the differences in vertical scales when comparing active versus inactive (left versus right) and crustal recycling versus embedded plume (top versus bottom) models.

We use the morphological differences in the gravity signals to complement topographic analysis of coronae, helping distinguish different tectonic scenarios and activity stages. We focus on free-air gravity anomalies as these display the most distinctive profiles across geodynamic scenarios. In addition, the calculation of Bouguer anomalies, both for the geodynamic models (fig. S4) and Venus data, involve inherent assumptions that complicate interpretation, such as a globally homogeneous crustal density value and choices made in interpolation between topography and gravity resolutions. The limited resolution in the current Venus gravity dataset restricts a detailed analysis (Fig. 4). The free-air gravity signals at typical Magellan resolutions (spherical harmonic degrees 70 to 90, corresponding to 270 and 210 km; see Methods) can only capture the amplitude of the gravity anomaly but not fine-scale details (Fig. 4). The same signals at the resolution predicted for the VERITAS mission, with an anticipated mean spherical harmonic degree better than 179 (<106 km) (*24*), would capture finer gravity signal morphologies (Fig. 4), highlighting the potential for improved gravity analyses in the near future.

**Fig. 5.**
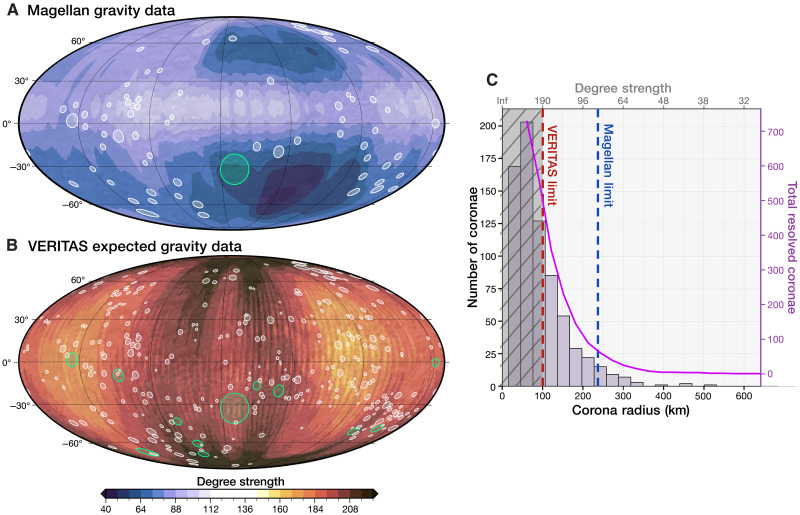
Resolvable coronae in the Magellan and VERITAS gravity datasets. (**A** and **B**) Distribution of (broadly/finely) resolved coronae superimposed over the Magellan global gravity degree strength (A) and the predicted VERITAS global gravity degree strength (B). For a definition and discussion of degree strength, see Methods. The maps are in Mollweide projection centered at 130°E. White features indicate broadly resolved coronae (one to three independent data points within the radius), while green features mark finely resolved coronae (four or more data points within the radius). See Methods for further details. (**C**) Histogram illustrating the distribution of coronae diameters [from (*5*)]. The purple solid line represents the cumulative count of coronae smaller than the specific diameter. The blue and red vertical dashed lines mark the average resolutions of VERITAS and Magellan of *l* = 190 and *l* = 80, respectively. The gray shaded area highlights coronae smaller than the best possible global average resolution that VERITAS can achieve.

We assess which of the 740 cataloged coronae (*5*) are resolved in the Magellan gravity field and calculate their free-air gravity anomaly compared to their immediate surroundings (see Methods). The spatial resolution of Magellan gravity data is determined from the degree strength [(*25*) and Fig. 5].

We define the spatial resolution as the full wavelength corresponding to the maximum gravity degree that is resolved locally (see Methods). In contrast, Johnson and Richards (*23*) defined spatial resolution using half a wavelength. Our more restrictive definition is motivated by the substantial impact of low resolution on coronae gravity signals, as demonstrated below.

We define three categories based on each corona’s topographic radius relative to the local gravity resolution: “finely resolved,” “broadly resolved,” and “unresolved” (see Methods and Fig. 5A). Only Artemis (132.1°E, 32.5°S) is “finely resolved,” meaning its topographic radius is at least four times larger than the local gravity resolution. Another 74 coronae are “broadly resolved”, with topographic radii equal to or larger than the local gravity resolution. The remaining coronae are unresolved (see Methods). Applying these resolution metrics for coronae differs from previous work (*23*), which analyzed coronae with clear gravity anomalies regardless of their size, as we aim to establish a metric for comparing the capabilities of different gravity datasets, as will be discussed below.

Of the 75 resolved coronae, we find that 52 have positive local free-air gravity anomalies, indicative of underlying diapir buoyancy (which we therefore term active). Of these, 96% also display negative local Bouguer gravity anomalies (table S1), consistent with an active plume underneath. Meanwhile, 17 coronae display negative free-air anomalies, and 6 show no substantial anomalies relative to the surrounding terrain (table S1 and Fig. 1A). Among active coronae also analyzed by (*23*), 80% fall under their “uncompensated” (active) category (see Methods and the Supplementary Materials). Most active coronae are situated over global geoid highs, whereas those in geoid lows exhibit absolute negative free-air gravity values but are locally positive anomalies (table S1). In terms of topography, most active coronae feature (partial) trenches and are either embedded within rift zones (*N* = 16) or appear as isolated features (*N* = 18) (Fig. 1A). Other active, isolated coronae feature raised rims and elevated interiors (*N* = 12). Those with negative free-air anomalies typically have elevated rims with diverse interiors (*N* = 12), aligning with relatively inactive phases in our models (Fig. 3). Some coronae with negative (*N* = 5) or indiscernible (*N* = 3) free-air gravity anomalies also exhibit partial trenches (Fig. 1A and table S1), a feature our geodynamic models predict only with active plume-induced crustal recycling.

We apply a newly designed fitting algorithm to representative coronae to identify the geodynamic scenarios that best match their Magellan topography and gravity (*25*, *26*) (see Methods). The algorithm first determines the best geodynamic fits based solely on topography and then refines the evolutionary stage by comparing the free-air gravity signals of these best-fitting geodynamic scenarios through time with the Magellan data.

### (Partial) plume-induced crustal recycling at coronae

**Fig. 6.**
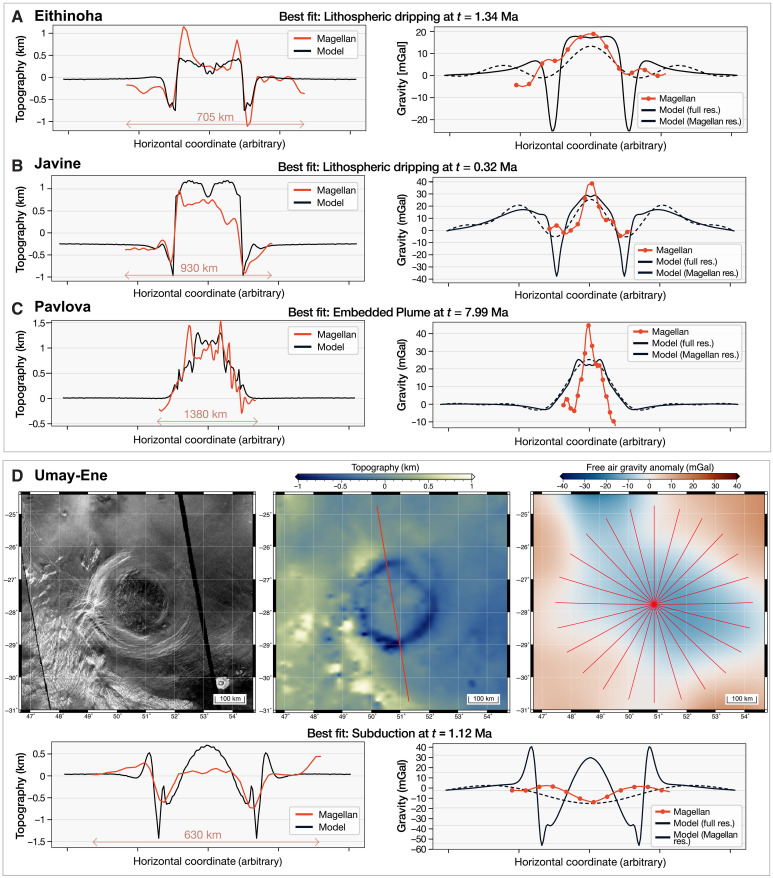
Summary of topography and gravity fitting results for four selected coronae. (**A** to **C**) Summary of fitting results for (A) Eithinoha (8°E, 57°S, full fitting results in fig. S5), (B) Javine (251°E, 5°S, full fitting results in fig. S7), and (C) Pavlova (40°E, 14.6°N, full fitting results in fig. S9), all considered to be overlaying active mantle plumes with various tectonic processes at play. The locations of these topographic profiles are shown in Fig. 1 (B and C). The left panels show the comparison between the best-fitting model topography and the selected Magellan topographic profile. In these panels, the original lengths of the observed Magellan profiles (in kilometers) are indicated by the red arrowed line. The original horizontal scale of the full geodynamic profiles is always 2000 km. The right panels show the comparison between the best-fitting model free-air gravity at full model resolution (black solid line), rescaled at Magellan resolution (black dotted line), and the observed Magellan gravity (red solid line). (**D**) Overview map of SAR imagery (left), topography (middle), and free-air gravity (with the best-fitting plane removed, right) for Umay-Ene (50.6°E, 27.7°S), with the fitting results given underneath. The maps have a cylindrical equidistant projection. More details on the fitting methodology are given in Methods.

The 16 coronae embedded within rifts (chasmata), marked by high free-air gravity anomalies coupled with topographic trenches, are plotted in blue in Fig. 1A. Many are within the BAT (Beta-Atla-Themis) rifts, while others are in different chasmata, such as Atahensik in the Dali-Diana chasma and Eithinoha near Lada Terra (Fig. 1, A and B). Eithinoha (8°E, 57°S) displays pronounced local high free-air and low Bouguer gravity anomalies. Figure 6A shows the best-fitting geodynamic model alongside observations (complete fitting results in fig. S5). The trench, rim, and relatively flat interior are typical topographic features of early-to-mid stages of plume-induced crustal recycling (Fig. 3, A and B), confirmed by the good match of the free-air anomaly. While the topography correlates highly with both lithospheric dripping and subduction, free-air gravity slightly prefers a lithospheric dripping scenario. We reach similar conclusions for Atahensik (170.6°E, 19.7°S), also with a pronounced local high free-air and low Bouguer gravity anomaly. Fitting results indicate ongoing plume activity and crustal recycling in its mid-to-late stage (fig. S6). Topographic fitting favors a mid-stage crustal recycling scenario as the mechanism at this corona; while the high free-air gravity anomaly shows a preference for a subduction mechanism, in line with studies suggesting retreating subduction at Atahensik’s southern margin (*22*, *27*). We note that our geodynamic models do not directly simulate rifting processes, which may be relevant for exploring coronae linked to rifts.

Another 18 isolated coronae exhibit local positive free-air gravity anomalies and (partial) topographic trenches (red features in Fig. 1A). Notable examples include Quetzatlpetlatl, Nightingale, Otygen, and Javine (table S1). Many of these coronae have been suggested to be tectonically active, indicated by topographic trenches (*15*, *27*), low estimated elastic thickness values (*2*, *28*), and/or free-air gravity analyses (*23*). Our fitting procedure for Javine (251°E, 5°S; Fig. 6B and fig.S7) reveals strong evidence of buoyant plume activity and positions the corona in the early-to-mid stages of plume-induced crustal recycling. The raised rim and interior surrounded by minor trenches fit reasonably well across all geodynamic scenarios from early to inactive stages (fig. S7). Gravity, however, refutes inactive embedded/underplated plume scenarios, favoring early lithospheric dripping followed by subduction. Similarly, our fitting procedure for Otygen (31.1°E, 57.2°S; fig. S8) reveals strong evidence of plume-induced crustal recycling in its mid-life stage.

### Embedded/underplated mantle plumes underneath isolated coronae

Twelve isolated coronae feature high rims surrounding elevated interiors with clear gravity anomalies suggesting plume activity. Many of these coronae—such as Pavlova, Ninmah, and Isong—are situated in the eastern Eistla Regio (Fig. 1A). For Pavlova (40°E, 14.6°N), our fitting procedure (Fig. 6C and fig. S9) consistently aligns with a noncrustal-recycling scenario. Topography alone favors a late stage of evolution of an embedded/underplated plume, while including gravity excludes inactive stages with high confidence. The high central free-air gravity anomaly (∼40 mGal) favors an earlier, active stage of the embedded plume geodynamic regime (Fig. 6C and fig. S9).

Similarly, for Aruru (262°W, 9°N; fig. S10), topography-only fitting slightly favors an inactive stage of embedded/underplated plume scenarios, while the strong positive free-air gravity anomaly (∼40 mGal) indicates an early evolution stage. These assessments align with studies identifying Aruru as active (*14*) and the eastern Eistla Region as a current “corona-dominated hot spot” (*19*, *29*).

### Potentially obscured gravity anomalies in the Magellan dataset

Although our geodynamic models do not predict coronae with topographic trenches combined with negative or indiscernible free-air gravity anomalies, we identify several such coronae on Venus (table S1). For instance, Demeter (295.2°E, 54.2°N; fig. S11) exhibits a topographic profile consistent with active plume-induced crustal recycling (fig. S11), previously considered evidence of ongoing plume-lithosphere interactions (*15*). However, Magellan free-air gravity reveals a notable negative central anomaly (−18.5 mGal), seemingly ruling out a buoyant mantle plume underneath. Demeter’s gravity profile is extremely well captured by later stages of Lithospheric Dripping case, but the fit to topography is relatively poor in this case (fig. S12), suggesting some degree of discrepancy between gravity and topography. Another example is Umay-Ene (50.6°E, 27.7°S; Fig. 6D and fig. S13), where the topography-only fit strongly prefers active tectonic recycling (fig. S13). The best gravity match suggests mid-life subduction despite the negative local gravity anomaly (table S1). This can be explained as an artifact of the Magellan gravity resolution (Fig. 6D and fig. S13). Although the full-resolution model predicts a positive local gravity anomaly at the corona center (and outermost rims), the low-pass filtering operated by the limited Magellan spatial resolution results in a negative central anomaly.

## DISCUSSION

Our work provides a general framework for identifying preferred plume-induced tectonic processes at coronae, and the results strongly suggest that a wide range of plume-lithosphere interaction processes are occurring across 52 coronae on Venus today (∼70% of resolved coronae). By systematically combining topography and gravity with geodynamic models, our study goes beyond previous studies that proposed which coronae are active based solely on comparing topography (*15*) or low-resolution topography and gravity (*14*) with geodynamic models or gravity alone (*23*). For most coronae, gravity data were important for identifying their most likely geodynamic regime and narrowing down their evolution stage. For instance, coronae with a raised rim and interior were often considered unclassified in (*15*) due to the absence of a narrow trench—typical of active crustal recycling. However, the topography and gravity displayed by these coronae (e.g., Pavlova, Fig. 6C and fig. S9) are typical of active embedded/underplated plume scenarios. This highlights the power of combining gravity and topography. Moreover, the demonstration of potentially obscured Magellan gravity anomalies for certain coronae (Fig. 6D) suggests that negative or indiscernible gravity anomalies associated with topographic features typical of active plume-lithosphere interactions (e.g., deep, narrow trenches) could be due to the resolution limitations of the Magellan data. This has implications for conclusions from previous Magellan gravity analyses of coronae, especially those evaluating peak free-air gravity values to suggest (in)activity (*23*), and highlights the potential for hidden signals in the data. Note that due to the fundamental assumptions behind our models, such as the absence of elastic rheology and simplified melting processes and initial setups (see Methods), we do not infer precise formation ages of coronae. Instead, we classify them in macro-evolution stages. Moreover, while our geodynamic models are radially symmetric, most coronae are not (e.g., Javine; fig. S7). Therefore, our inferred tectonic processes do not always represent fully symmetric dynamics [marked by “(p)” for partial in table S1]. We also cannot exclude the possibility that the analyzed coronae have formed via mechanisms outside our four geodynamic scenarios, such as the unexplored Rayleigh-Taylor instability scenario (*17*, *18*), or in in-between scenarios. In particular, coronae with contrasting topography and gravity fits, such as Demeter (figs. S11 and S12), may represent inactive stages with trench formation driven by loading or relaxation processes (*10*, *30*), although to-date, no existing geodynamic models captured narrow topographic trenches at final, inactive stages of evolution. Future geodynamic models that incorporate spatial scales, lithospheric properties, and tectonic settings specific to individual coronae will be invaluable in validating our study’s conclusions. Moreover, high-resolution topographic and gravity data, along with improved knowledge of Venus’ lithospheric structure, will allow for more individual assessments of coronae, enhancing our understanding of the planet’s global geodynamics.

### Future mission capability of revealing tectonic processes at coronae

The next decade will see multiple missions to Venus aimed at gathering updated and more accurate datasets. Notably, NASA’s VERITAS and ESA’s EnVision have among their scientific objectives the detailed mapping of the Venus gravity field (*24*, *31*–*34*). VERITAS, due to its low altitude and near-circular orbit, and two-way Ka-band tracking system will yield a gravity field resolution better than 106 km over >90% of the planet [degree strength 179, as defined as in (*25*)] with peaks at 85 to 90 km (degree strength 210 to 220) over specific areas (*24*, *35*) (Fig. 5B).

A VERITAS-like gravity dataset will provide an unprecedented level of detail compared to the Magellan gravity resolution (Fig. 4), which will enable focused analyses of tectonic/magmatic structures not possible until now. In this study, we used only the macro-characteristics of the gravity signatures of the analyzed coronae, but finer details will help categorize tectonic regimes and distinguish between different crustal recycling tectonics (lithospheric dripping versus subduction) (Figs. 3 and 4). Higher resolution gravity will also help resolve ambiguities in coronae showing topographic profiles typical of plume-induced tectonic activity but associated with currently negative or near-zero gravity anomalies. Moreover, more accurate inferences about the evolution stage of the corona may be made. While the Magellan free-air gravity anomaly maps are limited by a 9.7-mGal noise floor (global average) (*25*), the VERITAS gravity data will be much less noisy. With an expected noise floor of 0.5 mGal when gridded at 200 km (*24*), the VERITAS free-air gravity data will resolve smaller anomalies with greater certainty. Combined with geodynamic models tailored to specific coronae, this would allow for a more precise determination of corona evolution stages, potentially even revealing subtle signatures related to the early onset of new coronae or late-stage features.

With the degree strength map (*24*) for a VERITAS 2031 launch and nominal mission duration (∼2.7 years; see Methods) and using the same resolution classification for coronae as defined for Magellan data, we find that 415 coronae will be broadly resolved and 12 in greater detail (Fig. 5, B and C). This substantially advances current capabilities. Nine of the 12 expected finely resolved coronae are here classified as “active plume-induced crustal recycling”. This improved gravity resolution (≥4 data points per radius), together with other forthcoming data on Venus’ lithospheric structure, could enable the revealing of specific crustal recycling processes at these coronae. Sites of particular interest for VERITAS include not only Eithinoha, Atahensik, Javine, and Otygen for distinguishing recycling scenarios but also Demeter and Umay-Ene to confirm potential masking of local gravity anomalies by the poor Magellan gravity resolution. Overall, a better understanding of the spatial distribution of proposed shallow, thermal mantle plumes and sites of crustal recycling, combined with the lithospheric structure of Venus, will substantially contribute to understanding the interior processes that shape(d) Venus’ surface. Last, future research should investigate which coronae are expected to exhibit additional detectable forms of geological activity, such as lateral/vertical surface motion and volcanic outgassing, that can be directly measured by the instrumentation onboard the upcoming Venus missions. Together with topographic and gravitational signatures, this will guide the selection of regions of interest for these missions and provide valuable insights into Venus’ global geodynamic regime.

## METHODS

### Numerical technique and geodynamic model setup

Four end-member geodynamic models from (*15*) were run for a longer duration, analyzed, and post-processed for this study. The numerical experiments were executed using the staggered grid/particle-in-cell viscous-plastic 3D code I3ELVIS (*36*, *37*), designed to investigate the dynamics involved in plume-lithosphere interactions by incorporating a thermo-rheologically realistic lithosphere fully coupled to mantle dynamics in three dimensions. A full description of the code can be found in the Methods section of (*15*); here, we summarize only the major points related to the technique. I3ELVIS is a parallel implicit multi-grid code that combines the finite difference method, applied on a fully staggered Eulerian grid, with a marker-in-cell technique (*38*). The momentum, continuity, and energy equations are solved on the Eulerian grid, while physical properties are transported by Lagrangian markers that move according to the velocity field interpolated from the fixed grid. Non-Newtonian viscous-plastic rheologies are used in the model [extended data table 1 in (*15*)], and the code also accounts for mineralogical phase changes, adiabatic, radiogenic, and frictional internal heating sources. The model considers melt extraction and upward transport from the plume, rheological weakening of the lithosphere due to melt percolation, crustal growth through magmatic processes, and the basalt-to-eclogite phase change to capture essential geophysical plume-lithosphere interaction processes, as detailed in (*15*, *21*).

The models feature a spherical thermal mantle plume, 130 km in radius, situated beneath a basaltic crust, and a warm Venusian lithosphere [the reference model design is shown in extended data figure 2 in (*15*)]. The Eulerian domain spans 2020 km by 2020 km by 392 km, is resolved with a regular rectangular grid of 405 × 405 × 197 nodes, and contains 256 million randomly distributed markers. Boundary conditions include free-slip on all lateral sides and an open lower boundary to facilitate mantle flow. The lithospheric mantle, asthenosphere, and mantle plume share a dry olivine rheology, with visual differentiation to aid in observing dynamic processes. The initial thermal structure is based on an error function describing lithospheric temperature profiles (*39*), depicting a relatively warm and thin lithosphere consistent with recent theories about Venus’ lithospheric properties [e.g., (*40*)]. Detailed information on the specific setup, boundary conditions, and material properties essential for capturing the geodynamic processes is provided in (*15*).

### End-member geodynamic scenarios and active versus inactive stages

By systematically varying model parameters such as plume size and temperature, lithospheric strength, and crustal thickness, Gülcher *et al.* (*15*) identified four end-member regimes of plume-lithosphere interaction leading to large (>300 km in diameter) corona structures at the modeled surface. These four end members—lithospheric dripping, (ephemeral) subduction, embedded plume, and underplated plume—are investigated in detail in this work. Their evolutionary sequences are described in the Results and illustrated in Fig. 2. These end-member regimes largely depend on the relationship between plume buoyancy and lithospheric strength, as well as the crustal and lithospheric thicknesses (i.e., temperature at the base of the crust, Moho). Only a sufficiently buoyant mantle plume relative to the strength of the lithosphere can fully break apart the lithosphere and cause crustal recycling at its margins. For a warm and thick crust (high Moho temperature), dripping behavior is favored, while subduction is more likely for a cold and thin crust (lower Moho temperatures) or crust with stronger rheology. When the mantle plume is not buoyant enough to penetrate the lithosphere, it can instead remain embedded within the crust or underplated underneath (*15*).

The representative models for each geodynamic regime are: model M0 for lithospheric dripping (plume diameter of 130 km, temperature of 1888 K, lithospheric thickness of 45 km, and 30-km-thick crust), M9 for subduction (same parameters as M0 except for a thinner, 15-km-thick crust), M2 for embedded plume (same parameters as M0 except for a colder plume temperature of 1788 K), and M18 for an underplated plume (same parameters as M0 except for a stronger lithosphere through a lower effect of melt-induced weakening of lithospheric material). Each of these four geodynamic models is run longer than in (*15*), in which all models were run for 50 million years (Ma) to achieve stages we consider fully inactive. The models are determined to reach an inactive stage when subsurface thermal anomalies and tectonic activity cease, meaning insufficient lateral thermal anomalies (d*T*_*x*,*y*_ < 10 K) and mantle material velocities (*v*_*z*_ and *v*_*x*,*y*_ < 1 mm/year) underlying the surface topography (see inactive stages in Fig. 2). The crustal recycling scenarios (Fig. 2, A and B) achieve their final inactive stage within ∼ 100 Ma, much faster than the embedded/underplated plume scenarios (Fig. 2, C and D), which reach these stages around roughly 200 Ma. This is due to more efficient mantle cooling when lithospheric recycling occurs. The term active therefore refers to the period during which a thermal anomaly from the original plume or (partial) melt is present beneath the subsurface, causing topographic and gravity deflections until these thermal anomalies and tectonic processes at the plume margin cease.

### Model considerations

Several assumptions behind the geodynamic models should be considered for the geodynamic scenarios and the computed evolution speeds. First, the tectono-magmatic formulation used assumes instantaneous, vertical melt percolation toward the crust, which leads to an overestimation of the plume’s penetration speed through the lithosphere and prevents the development of flow field divergence in response to melt accretion at the base of the magma region. Lateral migration of melt could create localized melt ponds that support topography, as demonstrated in 2D two-phase flow models of plume-lithosphere interactions (*16*). Second, the visco-plastic rheological model of the lithosphere is simplified and does not account for rock elasticity, which would promote the development of more fractural features and likely affects the modeled topographic evolution. In particular, elasticity may dampen the high-amplitude topographies and allow the formation of trenches to persist for longer durations. It is, therefore, crucial to investigate 3D visco-elasto plastic models in the future to determine whether narrow trenches might be possible in “inactive” stages (*10*, *30*), i.e., for coronae with trenches but no gravity signature that indicates plume material. Last, plume activity beneath the surface could be prolonged and complexified by a constant or periodic supply of heat and magma to the coronae from multiple plumes, an active plume tail, or tectonically inherent features in the lithosphere (*21*).

### Deriving gravity anomalies from geodynamic models

The geodynamic models provide the evolving 3D distribution of, among many other fields, density, which is dependent on pressure, temperature, rock composition, and melt content (*15*). These density fields facilitate the analysis of the gravity signal. The total gravity signal, i.e., the free-air gravity, is a result of the density structure of the mantle and crust combined with the surface topography. We compute the free-air gravity of selected timesteps of each geodynamic model with a point-mass approach using the Python library Harmonica (*41*). At these selected timesteps, the density field from the geodynamic models is discretized into a regular grid (405 × 405 × 197), from which each element is treated as an independent point-mass source. We use a 300 × 300 observation grid placed on the top layer of the computational domain (i.e., *z* = 0), corresponding to a lateral spacing of 6.8 km, at which the gravity signal is calculated. The total gravity signal at each of these observation points is obtained by summing the contribution of each grid element.

Given the density field ρ(*x*, *y*, *z*) with *x*,*y*,*z* spatial coordinates defined on the Cartesian domain $X\times Y\times Z=\left[x_{0},x_{1}\right]\times \left[y_{0},y_{1}\right]\times \left[z_{0},z_{1}\right]$ and grid cell volume *V*, the vertical gravity acceleration felt by the point $P=\left[x_{p},y_{p},z_{p}\right]$ due to the density distribution ρ is computed as

$$g_{z}\left(P\right)=\sum_{x\in X}\sum_{y\in Y}\sum_{z\in Z}\frac{G\rho \left(x,y,z\right)V}{l^{3}}\left(z-z_{p}\right)$$ (1)

where *l* is the Cartesian distance between *P* and the density element (x,y,z). The free-air gravity anomaly is obtained from the total gravity signal by removing the normal gravity from the predicted gravity, i.e., by removing the gravitational acceleration of the unperturbed computational grid (i.e., using the far-field density profile in the absence of a plume/corona)

$$f\left(P\right)=\sum_{x\in X}\sum_{y\in Y}\sum_{z\in Z}\frac{G\left[\rho \left(x,y,z\right)-\tilde{\rho }(x,y,z)\right]V}{l^{3}}\left(z-z_{p}\right)$$ (2)

where $\tilde{\rho }\left(x,y,z\right)$ is the density grid associated with the unperturbed computational domain.

The Bouguer anomaly is computed by correcting the free-air anomaly for the topography [e.g., (*39*)]. For this correction, we assume a constant crustal density equal to 2900 kg/m^3^, which represents the effective density of the crust based on the models in (*15*). We note that this assumption does not account for possible density differences in the crust due to, e.g., the presence of melt or the effect of lateral temperature variations. The calculation is made according to

$$b\left(P\right)=f\left(P\right)-\sum_{x\in X}\sum_{y\in Y}\sum_{z\in Z}\frac{G\left[\rho^{*}\left(x,y,z\right)-{\tilde{\rho }}^{*}(x,y,z)\right]V}{l^{3}}\left(z-z_{p}\right)$$ (3)

where $\rho^{*}\left(x,y,z\right)$ and ${\tilde{\rho }}^{*}\left(x,y,z\right)$ equals 2900 kg/m^3^ if (x,y,z) is below the surface and 0 kg/m^3^ otherwise. $\rho^{*}$ is derived from the geodynamical model output, while ${\tilde{\rho }}^{*}$ is obtained from the unperturbed computational grid. Based on this equation and the assumptions involved, we note that even at inactive stages of the geodynamic models, small Bouguer gravity anomaly persists (see Fig. 3), as even at inactive stages, lateral variations in vertical temperature profile and hence effective interior density are present. The 2D fields of the free-air and Bouguer accelerations are obtained by computing Eqs. 2 and 3 over the observation grid [i.e., $f\left(P_{i}\right)$, $b\left(P_{i}\right)$ where $P_{i}$ are the observation grid points, and thus, $z_{p}=0$].

### Resolution of the Magellan gravity data

A commonly used metric to quantify the resolution of a scalar field expressed in spherical harmonics is the so-called degree strength. This quantity has been first defined to analyze the Magellan-derived gravity field of Venus (*25*) and is now widely applied in gravity-geodesy studies [e.g., (*42*–*46*)]. In short, the degree strength quantifies the local degree of the spherical harmonic expansion at which the signal-to-noise ratio is equal to one. At a surface point with degree strength $d_{s}$, features with a characteristic degree lower or equal to $d_{s}$ (larger wavelengths) are resolved, while those at a degree higher than $d_{s}$ (smaller wavelengths) are below the noise level. The degree strength map from the Magellan gravity field, calculated from the Magellan gravity dataset (Fig. 5A) and available on the Planetary Data System (PDS), is identical to the published map in (*25*) [see also the “Gravity a priori” section in (*25*) for detailed methodology on degree strength calculation]. We find an average degree strength of 75 ± 13 (mean ± SD). This value is area-weighted, meaning the degree strength maps are weighted as cos(θ), where θ is latitude, to avoid overweighting the poles when starting from a typical regular lat/lon grid.

The spatial resolution *L* needed to resolve a full wavelength is derived from the degree strength $d_{s}$ using the formula $L=\pi R_{\text{venus}}/d_{s}$, where $R_{\text{venus}}$ is the radius of Venus (6051.8 km). This formula stems from the fact that Legendre polynomials (the basis functions of spherical harmonics) of degree *l* have 2*l* zeros on the sphere. The degree strength derived from the Magellan gravity data is presented in Fig. 5A. Typical values of spherical harmonic degree strength range from 60 to 90, corresponding to spatial resolutions of approximately 315 and 210 km, respectively. Note that in the study on peak free-air gravity anomaly values within coronae (*23*), the Magellan gravity spatial resolution was defined as resolving half a wavelength (L=πR/2l) compared to the full wavelength as used in this study (L=πR/l).

### Coronae resolved in the Magellan gravity field

For our analysis of Venusian coronae, we use the recent database by (*5*), which documents a total of 740 corona features, alongside the global distribution of the Magellan gravity field’s spatial resolution. In (*5*), the diameter—twice the radius—of each corona is defined as the average distance between the outer fracture rims. However, a recent study by Sabbeth *et al.* (*47*) on coronae located within areas covered by the stereo-derived topography map from (*48*) indicates that the full topographical extent of these coronae typically exceeds the confines defined solely by fractures. Consequently, we adopt the topographic radius for our comparison of corona size to the spatial resolution of gravity data: $R_{\text{topo}}=1.2\cdot R_{\text{fractures}}$, with 1.2 being an expansion factor based on findings by (*47*).

Three classes are defined based on the comparison of corona topographic diameter and Magellan gravity field resolution: finely resolved, broadly resolved, and unresolved. A finely resolved corona is defined as one whose radius is at least four times the local spatial resolution of the gravity field. Within the Magellan data, only Artemis qualifies as finely resolved. Another 74 coronae meet the criteria for being broadly resolved, having radii equal to or greater than the local spatial resolution of the gravity field (Fig. 1). The remaining 665 coronae are categorized as unresolved because their radii fall below the local spatial resolution.

Our method of applying firm resolution thresholds slightly differs from that by (*23*), which analyzed all coronae with a peak free-air gravity anomaly, regardless of their size, and applied a resolution criterion only to establish if the absence of a signal at coronae with no discernible free-air gravity anomaly was related to gravity resolution or not. Moreover, because of the different definitions of Magellan gravity spatial resolution (half a wavelength versus full wavelength per resolved degree), more coronae with indiscernible anomalies would be considered resolved in (*23*) compared to our definition. These methodological differences—combined with variations in corona radii compared to gravity resolution and the availability of a new, updated corona database (*5*)—contribute to the discrepancy in the number of coronae classified in (*23*) (*N* = 166) versus our study (*N* = 75). Our decision to apply firm resolution criteria to all coronae is driven by the goal of comparing the capabilities of different gravity datasets. Furthermore, our use of a full wavelength for defining spatial resolution of gravity is motivated by the substantial impact of the low Magellan resolution on coronae gravity signals with strong lateral variations, as demonstrated in the main paper.

### Magellan gravity anomalies of resolved coronae

The detected free-air and Bouguer gravity anomalies for all 75 resolved coronae are listed in table S1. For each corona, we provide both the absolute values, averaged within the circular boundary defined by its central coordinates and radius, and the local anomalies relative to its immediate surroundings. This distinction is crucial because local high free-air anomalies can occur in regions with negative absolute anomalies, as seen, e.g., Ma (56.8°E, 22.5°S) and Bau (258.7°E, 52.9°N) coronae, detailed in table S1. In comparing observations with geodynamic models, we focus on local anomalies of each corona relative to its immediate surroundings. The local anomaly is calculated as the difference between the peak value within the circular corona and the average of the surrounding area, taken as an annulus surrounding the corona spanning from one to three times its diameter. The regional gravity trend (i.e., the best-fitting plane) is removed to minimize the influence of large-scale gravity trends. This is consistent with the fitting algorithm (see the “Summary of algorithm for fitting geodynamic models to Magellan data” section). To detect anomalous corona gravity signals, we set a threshold of the peak local anomaly of ±10 mGal for categorizing a signal as an “anomaly.” This threshold selection is slightly larger than the spatially-averaged vertical error of 9.4 mGal in the Magellan free-air gravity measurements (*25*). The regional gravity setting of each corona is cross-validated to confirm that the automatic algorithm is not misled by nearby geological features with strong local gravity signals. This step is critical for coronae near features such as montes or chasmata (rifts) with high local free-air gravity anomalies, which can influence the regional value and thus the local corona anomaly. Examples include Miralaidji (163.8°E, 13.9°S) and Selu (6.4°E, 42.2°S), marked by an asterisk * in table S1. Moreover, for several other coronae, our algorithm detects anomalies, but regional analysis refutes these due to the presence of local geological features with strong gravity signatures (** in table S1). Ultimately, the verification of gravity signals of these complex regions, alongside relatively subtle anomalies, will likely require more precise future datasets.

While we only report the gravity anomalies of coronae considered resolved in the Magellan gravity field, we note that some coronae below our defined resolution threshold could still have a detectable (but smoothed) anomaly if they have a sufficiently strong signal. Based on our gravity anomaly algorithm alongside visual inspection, the following 19 unresolved coronae show a substantial, localized, positive free-air gravity anomaly compared to their surroundings despite their small dimensions relative to the gravity field resolution: Tamfana (6.1°E, 36.5°S), Tonatzin (164°E, 53°S), Earhart (136.7°E, 70.2°S), Khabuchi (173.2°E, 10.8°S), 51N52W (308.3°E, 51.2°N), Ukemochi (296.3°E, 39°S), Beyla (15.8°E, 26.3°N), Shulamite (284.4°E, 38.7°S), Fakahotu (107.1°E, 59.2°N), Gashan-Ki (243.7°E, 11.8°N), Hulda (308.2°E, 14.3°N), Sappho (15.7°E, 14.3°N), Benten (40.3°E, 14.6°N), 2N124W (236.4°E, 1.8°N), Tunehakwe (303.7°E, 33.3°S), Carpo (3.3°E, 37.5°S), 22N136W (224°E, 21.8°N), 19N133W (227.3°E, 19.3°N), Tefnut Mons (303.5°E, 38.3°S), and Kunhild (80.2°E, 19.4°N). Most of these coronae were considered uncompensated in (*23*), and their strong, localized positive free-air gravity anomaly is suggestive of a hot thermal anomaly below.

### Summary of algorithm for fitting geodynamic models to Magellan data

**Fig. 7.**
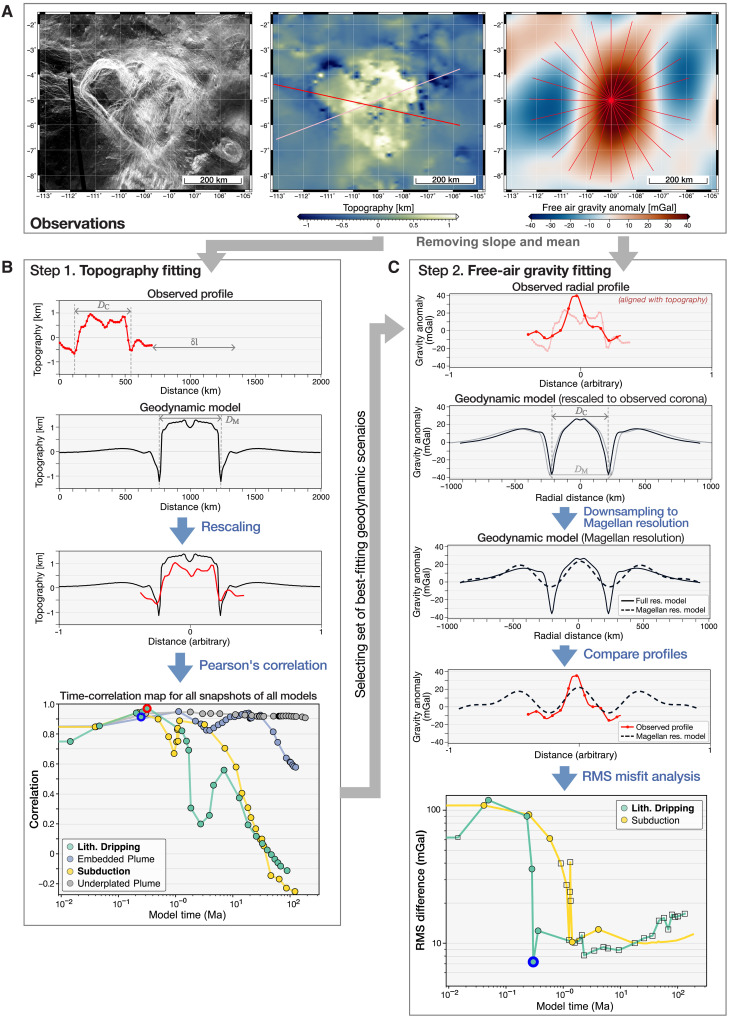
Summary overview of newly designed fitting algorithm for comparing observations to geodynamic models. Javine (251°E, 5°S) is used as an example to summarize the topography [step 1, (**B**)] and gravity [step 2, (**C**)] fitting algorithm. Profile 2 of Javine (right hand side in fig. S7) is used for this example. (**A**) shows the SAR, topography and free-air gravity of Javine (left, center, right). The red lines show the profiles used in our analysis. For details on the symbols and markers see the caption of fig. S5.

To leverage the computed output of the geodynamic models for identifying the optimal match to the Magellan data, we implemented a systematic fitting scheme that comprises two primary steps: (i) topography-only fitting and (ii) refined model selection based on free-air gravity fits. The steps involved are illustrated in Fig. 7. This scheme acknowledges that topography is resolved much more accurately than gravity in the Magellan datasets (typical horizontal resolutions of 10 versus 250 km, respectively). In summary, the fitting algorithm first uses topography to select the relevant geodynamic end-member scenarios for each corona and loosely restrict the evolution timestep range. It then refines this selection using gravity data to narrow down the preferred evolutionary stage of the corona. The details behind these steps are given below.

### Step 1. Identifying geodynamic scenarios best fitting to observed corona topographies

#### Selection of two representative topographic profiles of observed corona

The initial step involves identifying the geodynamic models whose topography most closely correlates with the observed Magellan topography (Fig. 7B). To achieve this, we manually selected profiles that capture the primary characteristics of each corona while avoiding any influence from extraneous geological features such as rifts and/or corona asymmetry. For example, for the asymmetric Javine (251°E, 5°S), a triangular-shaped corona with topographic trenches at two of its three major arcs, the profile was taken such that a topographic trench is present on both ends of the profile (Figs. 1C and 6B). For each corona, we chose two representative topographic profiles. To enable a good comparison with the model output (which is on an initially flat terrain), we removed the average value and slope from the observed topographic profiles. Because we are interested in the short-wavelength characteristics, the removal of long-wavelength characteristics (mean and slope) simplifies the analysis while not biasing the results.

#### Rescaling the topographic profiles of observations and models

To simplify the analysis and avoid complex geographic transformations, we scaled the radial axis of the modeled topographic profiles to range between −1 and 1. Because of the vertical grid resolution and the “sticky air” method of the geodynamic model (see above), its topographic surface is locally noisy on a 100-m level. Therefore, we used a radially averaged profile for our analysis. The observed topographic profiles cannot be scaled to the same radial range of −1 to 1 unless the dimensions of the modeled and observed corona topographies are identical. Therefore, we adjust the horizontal extent and radial position of the observed corona topographic profile by applying a “stretch” (*s*) and a “shift” (δ*l*) factor. The stretch parameter *s* adjusts the observed profile by either widening or shortening it to account for discrepancies in the diameters between observed and modeled coronae. The shift factor enables horizontal adjustments of the profile to minimize potential misalignments. These adjustments aim to maximize the Pearson’s correlation index P(m,d) between the modeled (*m*) and observed (*d*) corona topography profiles. More explicitly, given the observed topographic profile *d*(*x*), we incorporate the stretch and shift factors as follows

$$\tilde{d}\left(x\right)=d\left[s\left(x+\delta l\right)\right]$$ (4)

and seek to solve

$$\underset{s,\delta l}{\text{arg}\;\text{max}}P\left(\tilde{d}\left(x\right),\hat{m}\left(x\right)\right)$$ (5)

where $\hat{m}\left(x\right)$ represents the model topography interpolated to match the resolution of $\tilde{d}\left(x\right)$. We focused on maximizing the Pearson’s correlation rather than minimizing the root mean square (RMS) misfit because our primary goal is to capture the main morphological characteristics, such as trenches and rims, rather than achieving a perfect fit. This maximization procedure is executed for each timestep of every geodynamic model, culminating in a detailed map of maximum correlations. We use the Powell method implemented in scipy (*49*, *50*), which is a minimization algorithm. Thus, we solve

$$\underset{s,\delta l}{\text{arg}\;\text{min}}{\left\{P\left[\tilde{d}\left(x\right),\hat{m}\left(x\right)\right]\right\}}^{-1}$$ (6)

An example of the rescaled observed topography of the Javine versus a modeled profile for a single timestep is given in Fig. 7B. As the Magellan gravity resolution limits our analyses to the largest coronae on Venus, the size between observed and modeled coronae are roughly similar (*s* between 0.5 and 2.1). This avoids excessive rescaling of the output of the geodynamic model, which would lead to nonrealistic results.

#### Time-correlation maps

The output of this first step of our algorithm is a time-correlation map, which summarizes the correlation between the observed corona topography profile and the modeled topographies for all four geodynamic models at the timesteps for which gravity has been computed (Fig. 7B). By examining these correlation outcomes alongside the morphological features of the coronae, such as the presence or absence of trenches, we can distinguish between crustal recycling and noncrustal recycling scenarios. For instance, the topographic correlation results often favor subduction or dripping scenarios when distinct trenches are present in the topography. We isolate the set of most likely geodynamic scenarios, i.e., subduction/lithospheric dripping versus embedded/underplated plume, and proceed to the second step of our procedure, which integrates gravity data. This downselection of geodynamic scenarios for the gravity fitting algorithm is performed to retain the most substantial topographic information, specifically whether the observed topography is consistent with a crustal recycling or non-recycling scenario.

### Step 2: Comparing modeled and observed gravity anomalies to find the favored geodynamic snapshot 

#### Selection of average gravity profile of observed corona and alignment with the selected topographic profile

In the second step of the fitting algorithm, we compute the RMS difference between the free-air anomaly values from the data and the model for each timestep of the selected geodynamic scenarios (either lithospheric dripping/subduction or embedded/underplated plume cases) (Fig. 7C). To obtain a representative central free-air anomaly value and reduce the potential impact of geophysical “noise” from nearby geological features, we establish 12 linear profiles centered at the corona’s central coordinates [from (*5*)], spaced equally in azimuth, and average them. Before averaging, we eliminate the mean value and the average slope of the gravity, aligning with the method used to analyze the local gravity anomalies of coronae (table S1). This detrending simplifies the comparison of observed gravity data with geodynamic model outputs, which are inherently relative to a 0-mGal flat background. The resulting Magellan gravity profile’s horizontal coordinate is rescaled to match the observed topographic profile after alignment with the model size. Meaning that the gravity profile is converted to the same [−1, 1] domain of the topography.

#### Rescaling geodynamic modeled gravity to observed corona size and Magellan resolution

To compare the computed synthetic gravity anomalies with observed gravity data, we need to match the observed corona dimension and the local gravity data resolution. Initially, gravity anomalies from the geodynamic model—a 2D grid—are mapped onto a sphere and resized to match the actual dimensions of the observed corona. This involves calculating the ratio *I*_r_ between the observed and modeled corona radii and adjusting the modeled horizontal dimensions accordingly. The observed radius for a specific corona is sourced from (*5*), whereas the modeled radii for each model and timestep are automatically detected using a Hough circle transform implemented in the OpenCV library (*51*). This automated method analyzes the 2D gradient of the topography to identify the steepest gradients; these typically correspond to the deepest point of a trench in models with narrow trenches, the highest point of the rim in rimmed profiles without trenches, and the end of the dome in dome-shaped coronae. These locations indeed coincide with the locations of fracture rims (*47*), aligning with the radius definitions in (*5*). We conducted manual verification to confirm the accuracy of the detected corona radii across all examined geodynamic models. In all our fitting procedures, the scaling ratio *I*_r_ varies from 0.51 to 2.1, often resizing larger modeled coronae—particularly from crustal recycling scenarios—to fit observed counterparts (Fig. 3). Smaller coronae in the models, typically produced by smaller plumes, would require additional adjustments in lithospheric strength to maintain the plume buoyancy to lithospheric strength ratios and hence geodynamic regime (*15*, *21*). Consequently, comparable topography and gravity amplitudes may be expected, although further verification of this is needed in future studies. While this methodology does not ensure self-consistency in the resulting gravity signal or the inferred lithospheric properties, it is sufficient for identifying general geodynamic scenarios by analyzing trench presence, gravity anomaly nature, and morphology (*15*, *21*). After resizing the model 2D gravity field, we performed an exact spherical harmonic expansion of the full-resolution model (here we used a *l* = 2000 spherical harmonic expansion, although formally an exact expansion would require *l* → ∞). We then truncated the expansion at the degree corresponding to the degree strength of the specific location in the Magellan gravity field, and subsequently, we converted the truncated expansion back to a spatial representation using an antitransform. This procedure provides a realistic degradation of the model resolution, consistent with the spherical harmonic representation used by Magellan to measure Venus’ gravity.

#### Selection of best-fitting geodynamic scenario and time based on gravity

We interpolated the geodynamic model profiles to the Magellan profiles resolution and computed the RMS gravity difference between the modeled and observed profiles. We define the best-fit model as the one having the minimum RMS difference subject to the constraint of having a topographic correlation in the highest 20th percentile (i.e., a correlation value above the 80th percentile for each geodynamic model under evaluation). This way we retain the information coming from the topography fitting (we consider only the most likely models from the topographic standpoint) and use the gravity information to refine the model selection. The resulting model is the best-fitting case used for later discussion and interpretation (Fig. 7C).

Our rationale for selecting two distinct profiles for topography fitting while using a radially averaged profile for gravity anomalies, is motivated by the substantial resolution difference between the Magellan topography and gravity datasets (approximately 15 and 250 km in lateral direction, respectively). Given that topography is well resolved at a large corona’s length scale of hundreds of kilometers, using multiple topographic profiles could result in unrealistic smoothing, especially since most coronae exhibit strong topographic asymmetry (*21*, *47*, *52*). In contrast, averaging the gravity data is beneficial as it effectively reduces background noise and intrinsic error margins.

### VERITAS mission and the expected gravity field spatial resolution

NASA’s VERITAS mission (*31*) is a partnership led by NASA/Jet Propulsion Laboratory (JPL) between US scientists and engineers, with strong collaborations and contributions of the German, Italian, and French Space Agencies. On 2 June 2021, NASA selected VERITAS as one of the two winners of the Discovery 2019 competition. The launch is expected in 2031. Thanks to its orbital design, VERITAS will be able to completely map the planet every 243 days (one mapping cycle). The orbit will be kept stable (in the science phase 2, when the gravity science measurements will be collected) in a nearly circular polar orbit (180 km by 255 km in altitude, ∼85.4 inclination, and period of ∼1.5 hours). Such a low altitude allows for a very high sensitivity to small-scale variations in the gravity acceleration on the probe. Further, the two-way coherent Ka-band radio tracking provides greater precision than the X- or S-band data [e.g., (*53*–*55*)]. This results in a highly resolved global gravity field measurement. The expected quality of the VERITAS-measured gravity field of Venus is unprecedented, and several studies have shown how such an improved sensitivity will enable fundamental investigations into Venus’ interior structure [e.g., (*24*, *32*, *33*, *56*)]. For Magellan, the average degree strength is 75 ± 13 (mean ± SD), while for VERITAS, the expected average degree strength will be 197 ± 11, an improvement in spatial resolution of almost 300%. The expected global distribution of the degree strength of the VERITAS gravity field is given in Fig. 5B.

## Supplementary Materials

### This PDF file includes:

Figs. S1 to S13 Legends for data S1 to S3 Table S1 References
