# Supplementary material for: A spectrum of tectonic processes at coronae on Venus revealed by gravity and topography
Source: Sci Adv. 2025 May 14;11(20):eadt5932. doi: 10.1126/sciadv.adt5932 (PMC12077499; doi:10.1126/sciadv.adt5932)
Supplement: Supplementary file 1 — Figs. S1 to S13 Legends for data S1 to S3 Table S1 References [file sciadv.adt5932_sm.pdf]

Science Advances
AAAS

Supplementary Materials for

A spectrum of tectonic processes at coronae on Venus revealed by gravity and topography

Gael Cascioli *et al.*

Corresponding author: Gael Cascioli, gaelc@umbc.edu

Sci. Adv. **11**, eadt5932 (2025)
DOI: 10.1126/sciadv.adt5932

The PDF file includes:

Figs. S1 to S13
Legends for data S1 to S3
Table S1
References

Other Supplementary Material for this manuscript includes the following:

Data S1 to S3

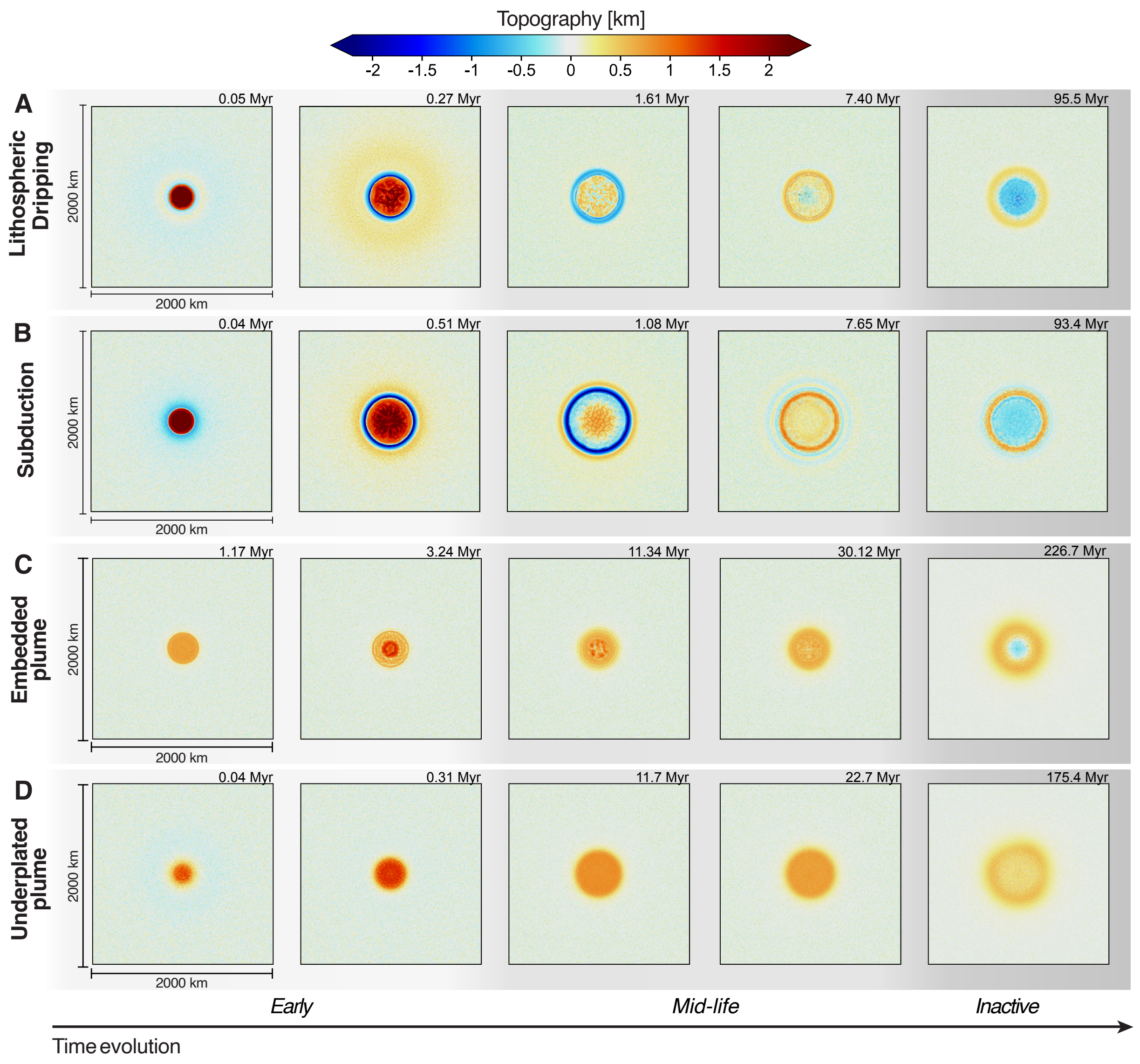

Figure S1: **Surface topography of geodynamic models through time**. Five snapshots are shown for each geodynamic end-member model (the same snapshots as Figures S2 and S3, also covering those in the main paper): **(A)** Lithospheric dripping, **(B)** Short-lived subduction, **(C)** Embedded plume, and **(D)** Underplated plume, as described in the main paper. In all models, the mantle plume initially rises, causing an elevated, circular surface (see left panels), but their subsequent topographic evolution is vastly different. For a description of the four geodynamic regimes, see the main text and Methods.

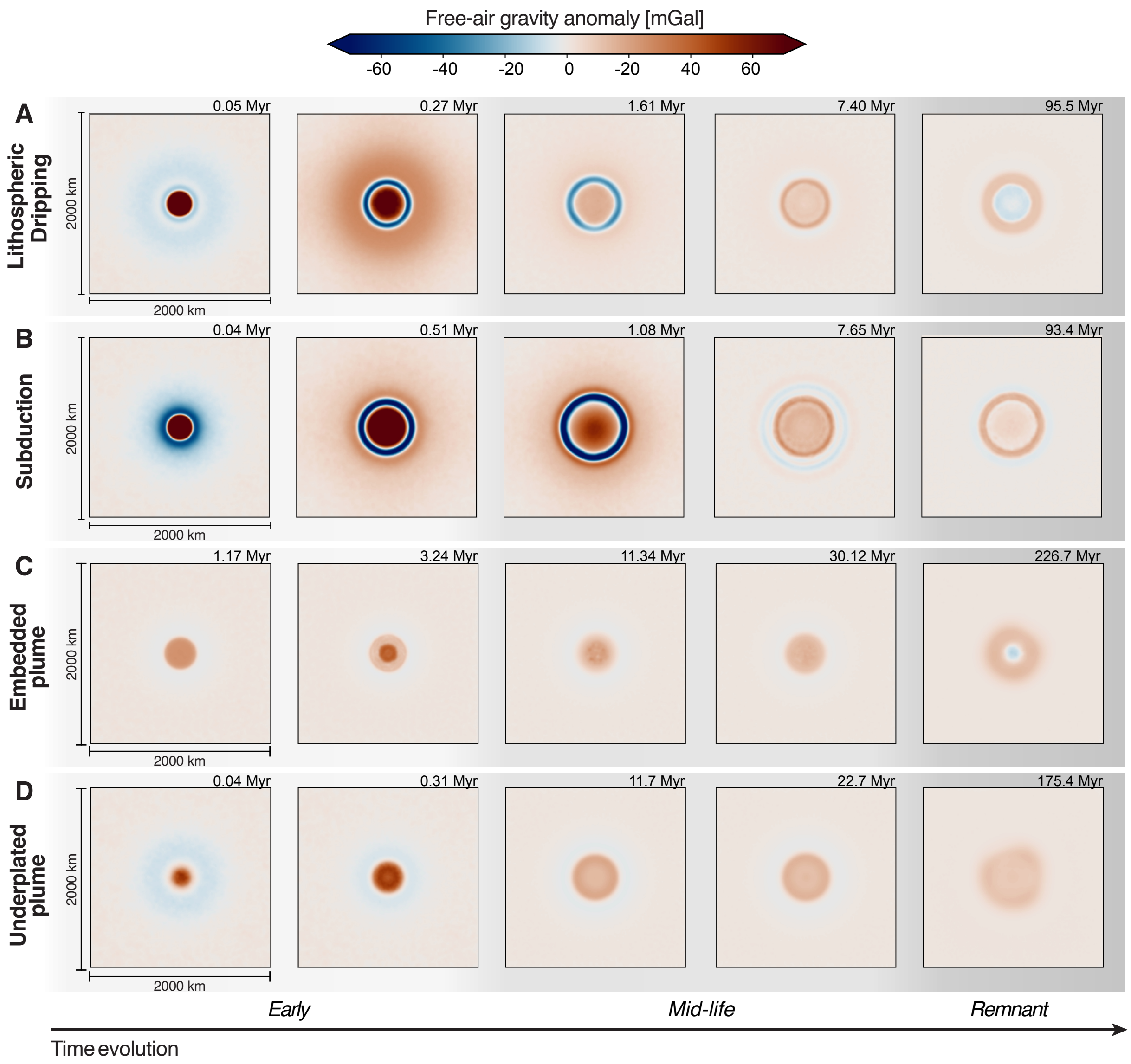

Figure S2: **Free air gravity anomaly of geodynamic models through time**. Five snapshots are shown for each geodynamic end-member model: **(A)** Lithospheric dripping, **(B)** Short-lived subduction, **(C)** Embedded plume, and **(D)** Underplated plume.

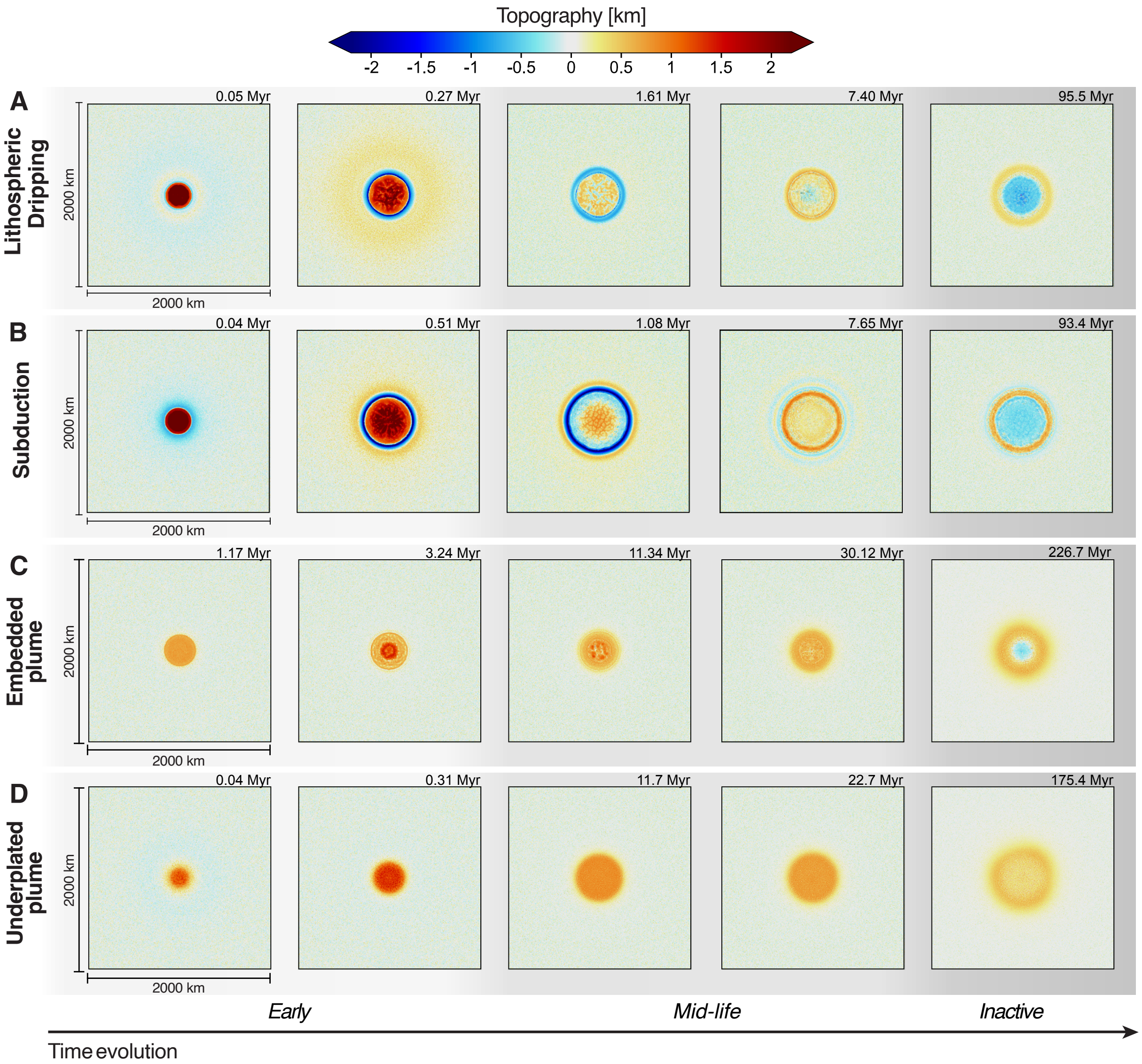

Figure S3: **Bouguer-corrected gravity anomaly of geodynamic models through time**. The same five snapshots as in figure S2 are shown for each geodynamic end-member model: **(A)** Lithospheric dripping, **(B)** Short-lived subduction, **(C)** Embedded plume, and **(D)** Underplated plume.

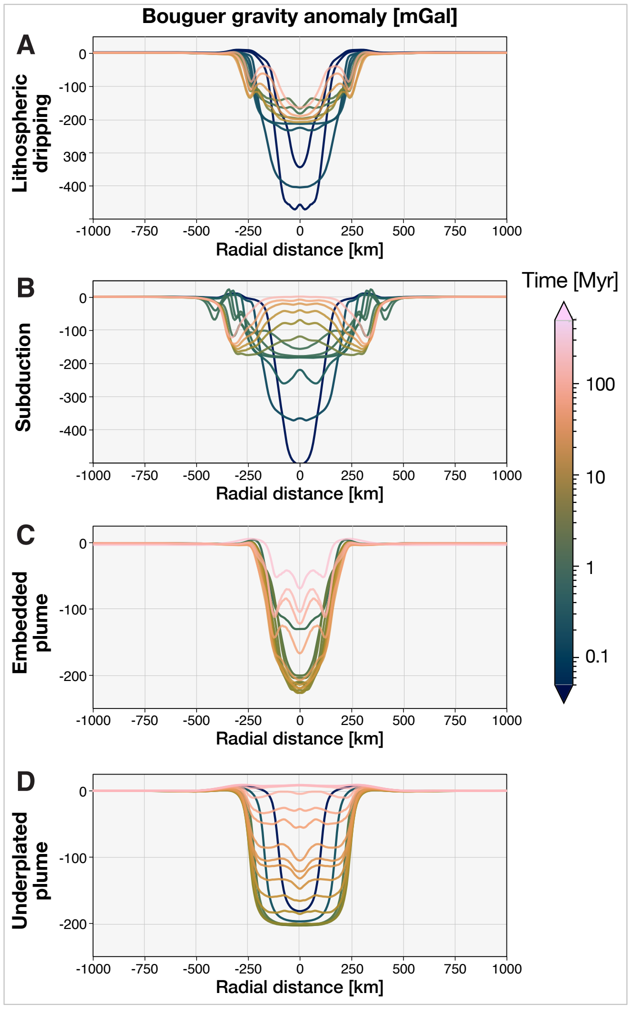

Figure S4: **Temporal evolution of radial Bouguer gravity anomaly of geodynamic models**. The panels show the evolution of the radially-averaged profiles in Bouguer gravity anomaly for the four geodynamic end-member cases: **(A)** Lithospheric dripping, **(B)** Short-lived subduction, **(C)** Embedded plume, and **(D)** Underplated plume, see main paper for more details. Note the different vertical scales for the crustal recycling scenarios (dripping/subduction, panels A and B) and the embedded/underplated plume cases (panels C and D).

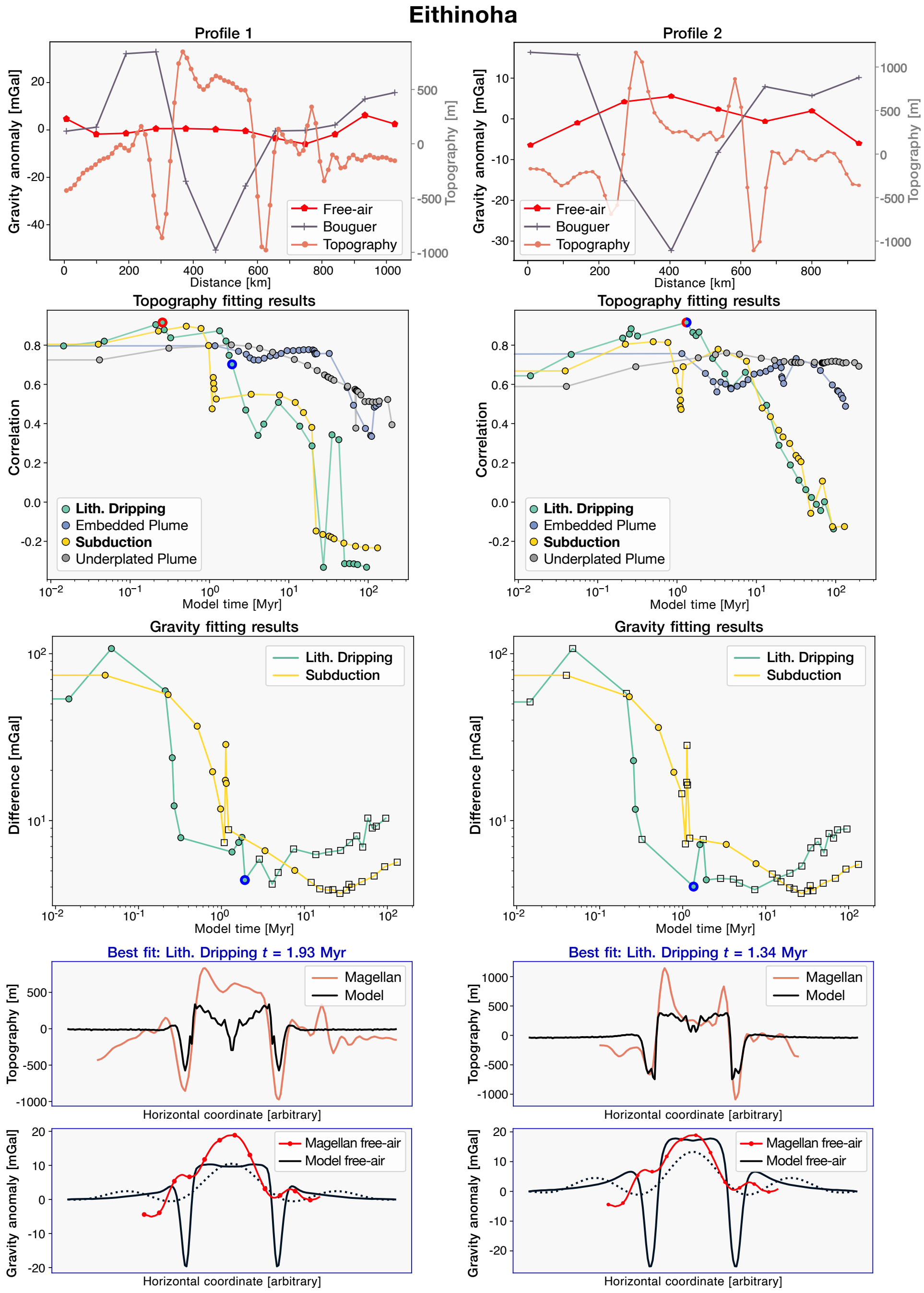

Figure S5: **Topography and gravity fitting results for Eithinoha** (8**E**, 57**S**)**.** Two linear profiles were selected (left and right columns). The second profile (right column) is also shown in Figure 6A in the main paper. first row provides overviews of the profiles in terms of topography and gravity anomaly. The second row shows the best correlation for each model and timestep. Red highlights the model with the maximum correlation based on topography-only fitting, and blue indicates the best fit after gravity comparison. The third row shows the RMS difference between the modeled and observed gravity, with squares representing models outside the top 20th percentile of topography correlation, and dots indicating models within it. The fourth row compares the topographic profiles of the best-fitting geodynamic model with the Magellan topography. The final row compares the free-air gravity anomaly of the best-fitting geodynamic model with the Magellan dataset (note that the Magellan free-air anomaly profile used in the fit is radially averaged as defined in Methods, while the first row shows the specific profile linked to the topography). Solid and dashed lines represent the full resolution and downscaled geodynamic models, respectively, with downscaling done at the Magellan resolution in the corona-specific region.

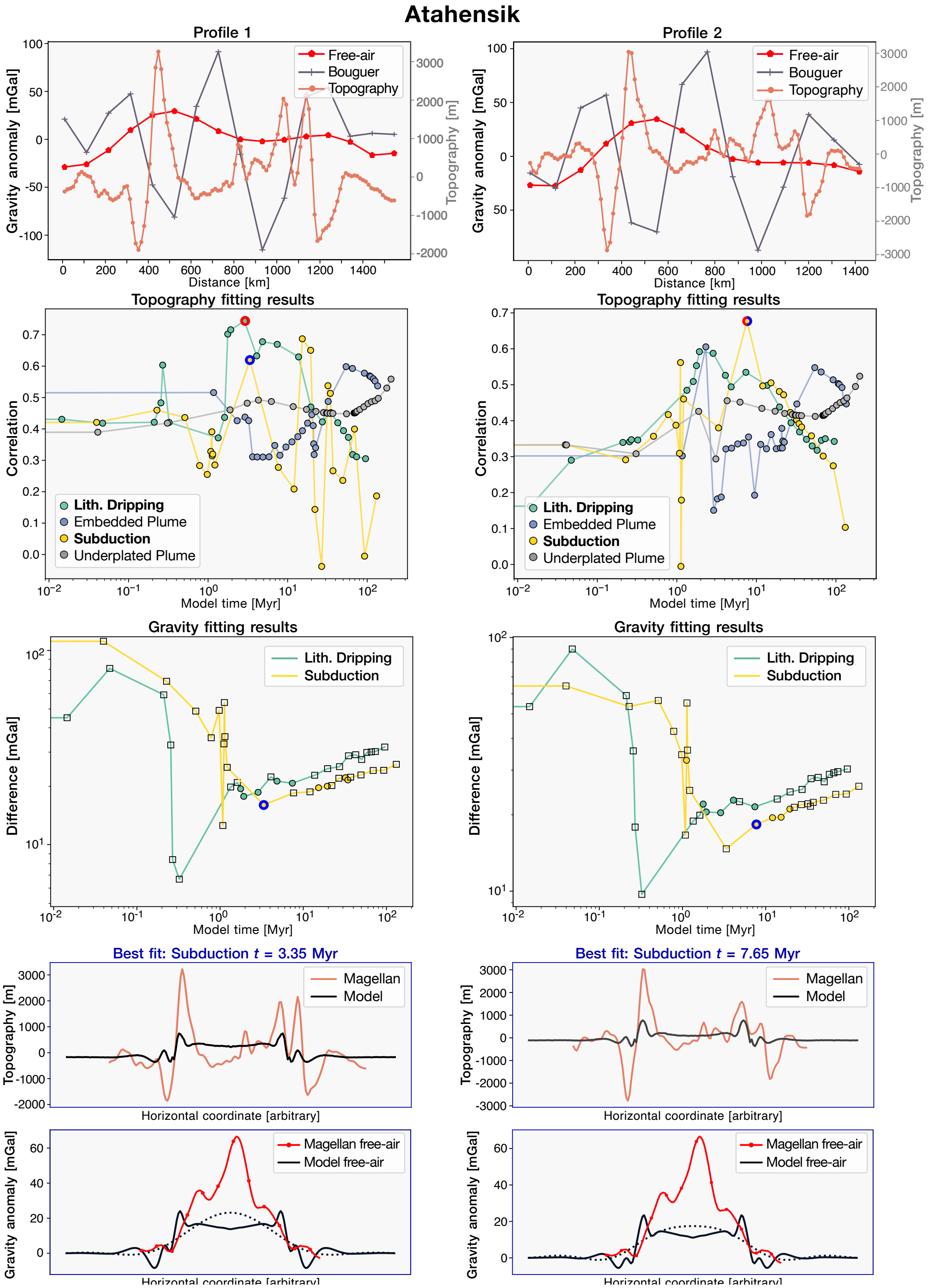

Figure S6: **Topography and gravity fitting results for Atahensik corona** (170.6**E,** 19.7**S).** The figure description is the same as in figure S5.

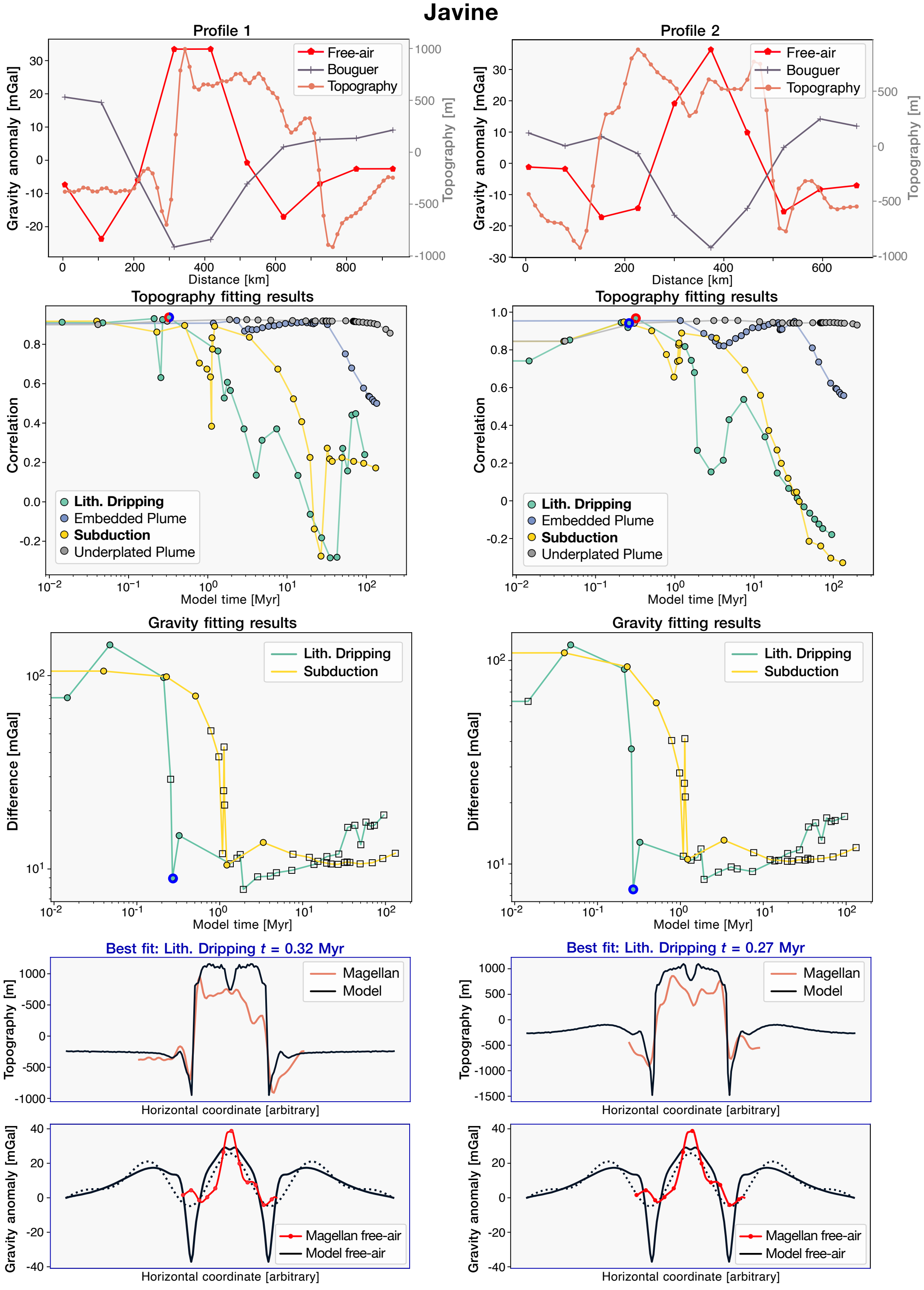

Figure S7: **Topography and gravity fitting results for Javine corona** (251E, 5S). The first profile (left column) also shown in Figure 6B in the main paper. The further figure description is the same as in figure S5.

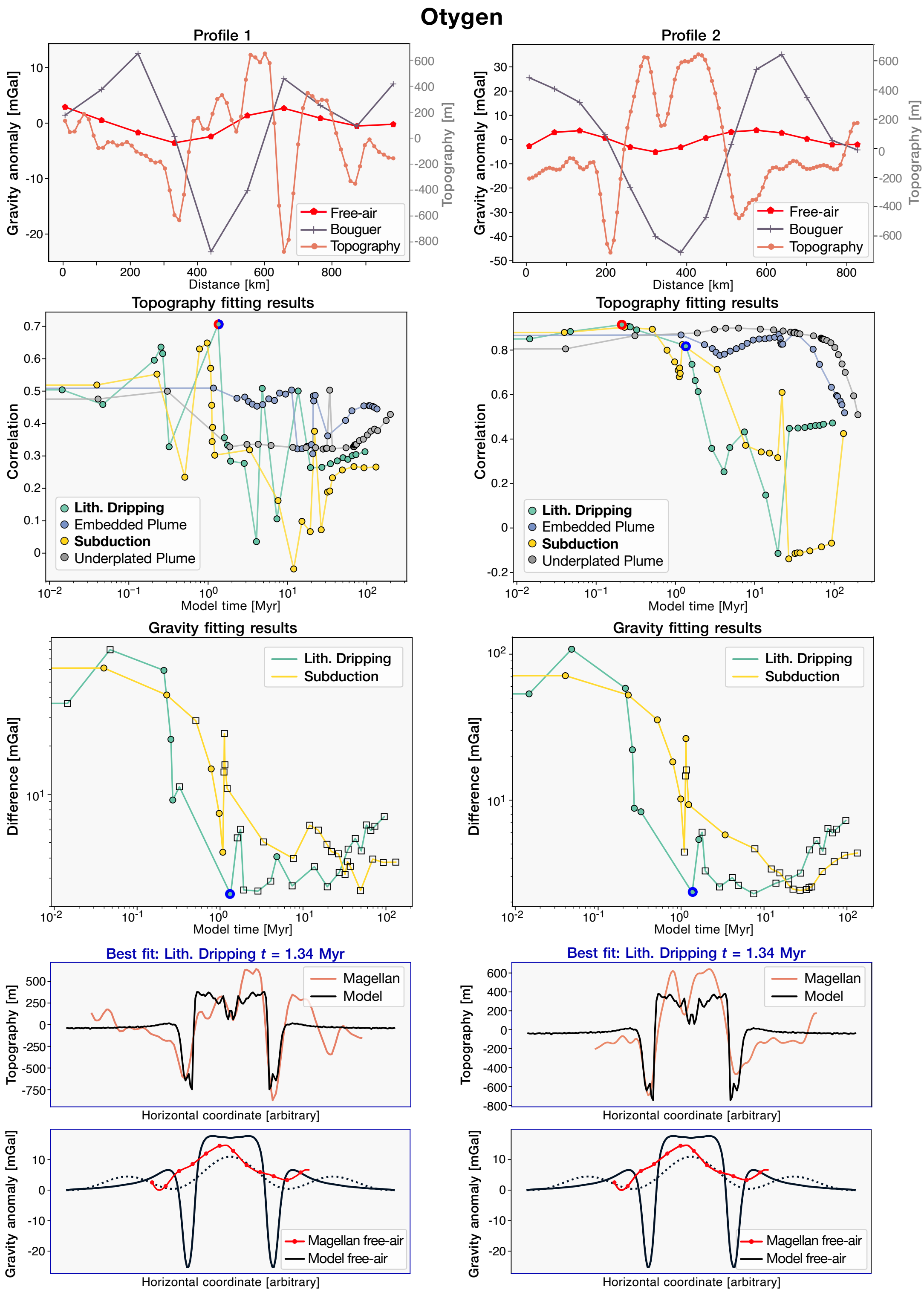

Figure S8: **Topography and gravity fitting results for Otygen corona** (31.1**E**, 57.2**S**). The figure description is the same as in figure S5.

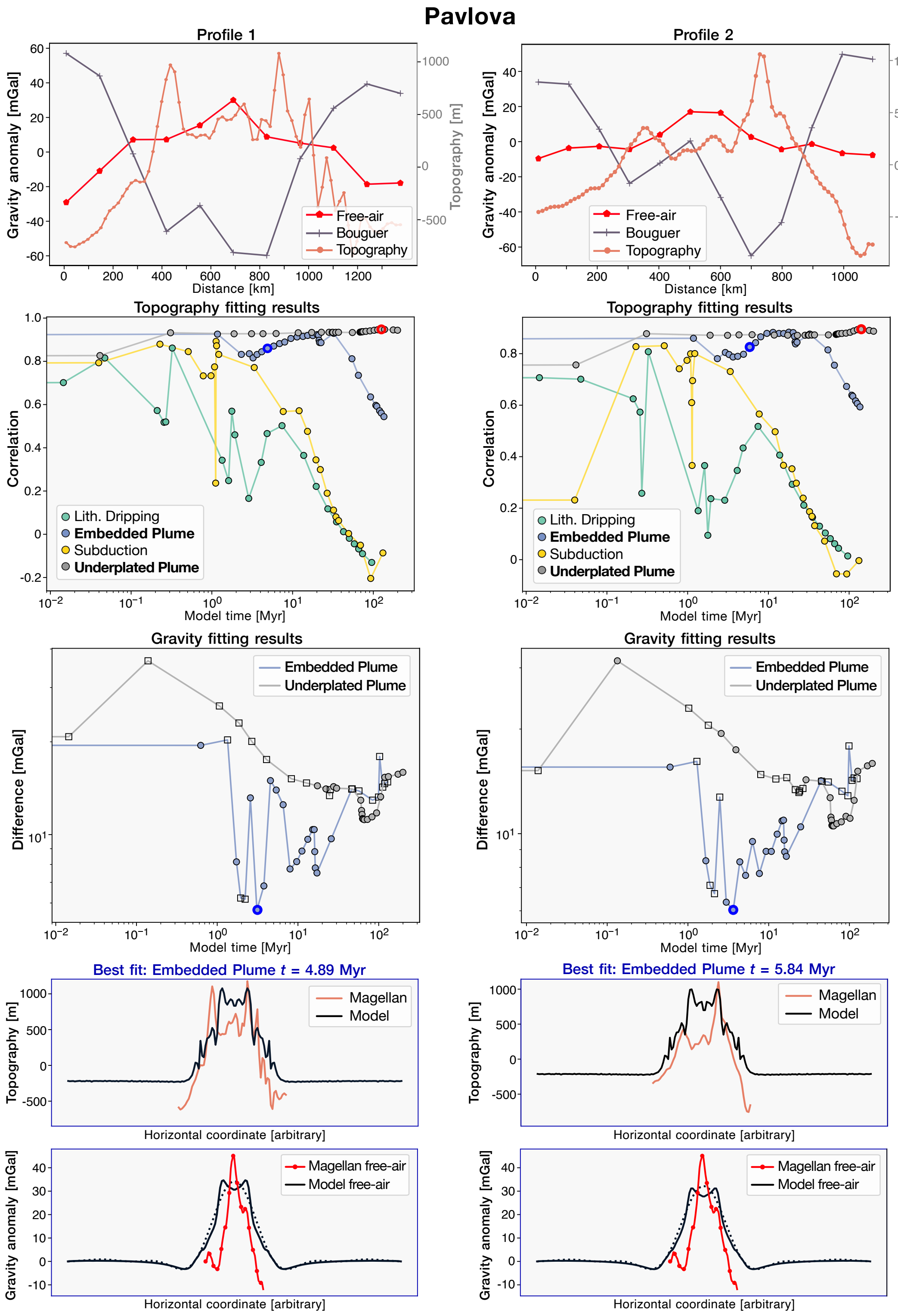

Figure S9: **Fitting results for Pavlova** (40**E,** 14.6**N**) The first profile (left column) also shown in Figure 6C in the main paper. The further figure description is the same as in figure S5.

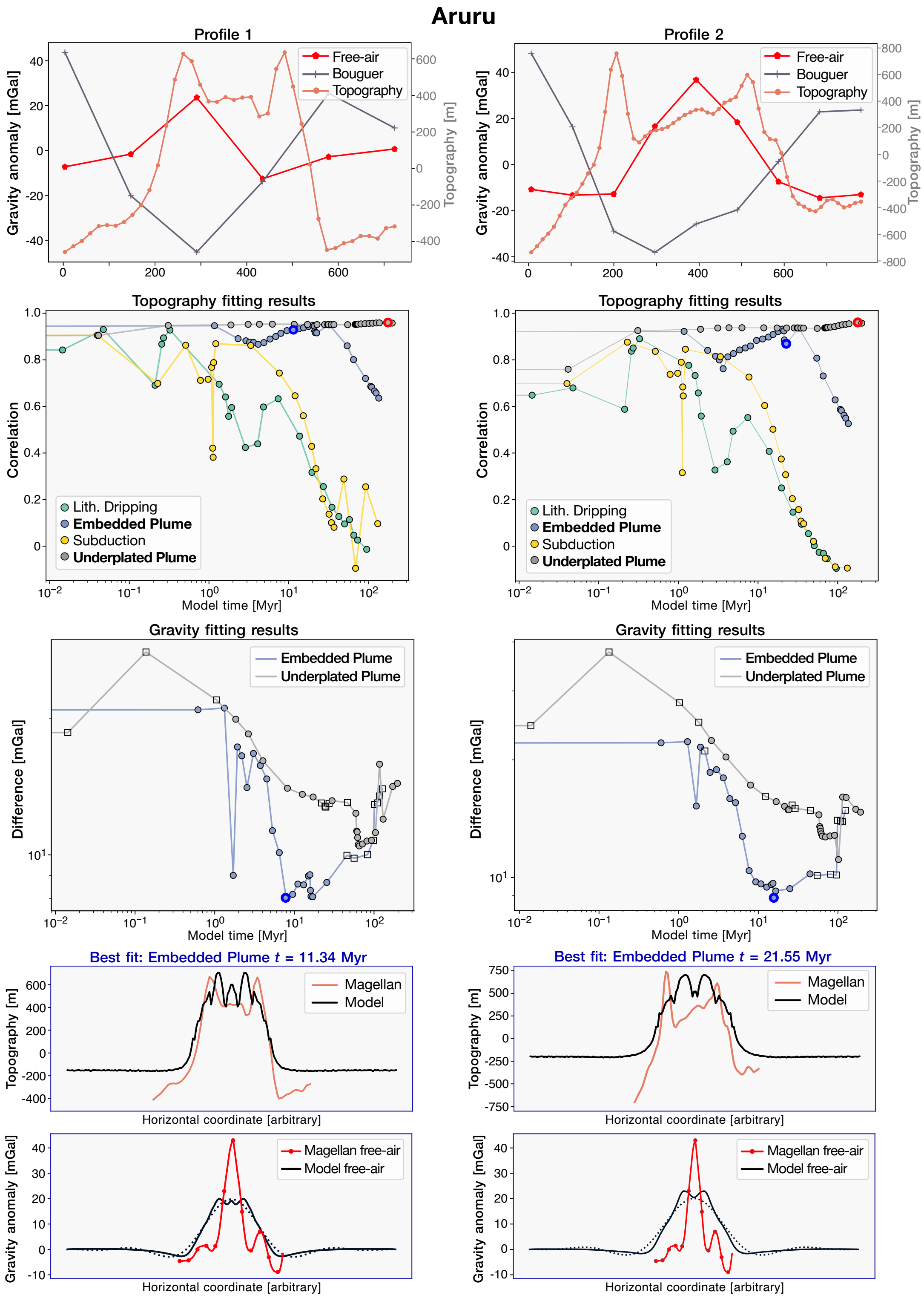

Figure S10: **Topography and gravity fitting results for Aruru corona** (262E, 9N)**.** The figure description is the same as in figure S5.

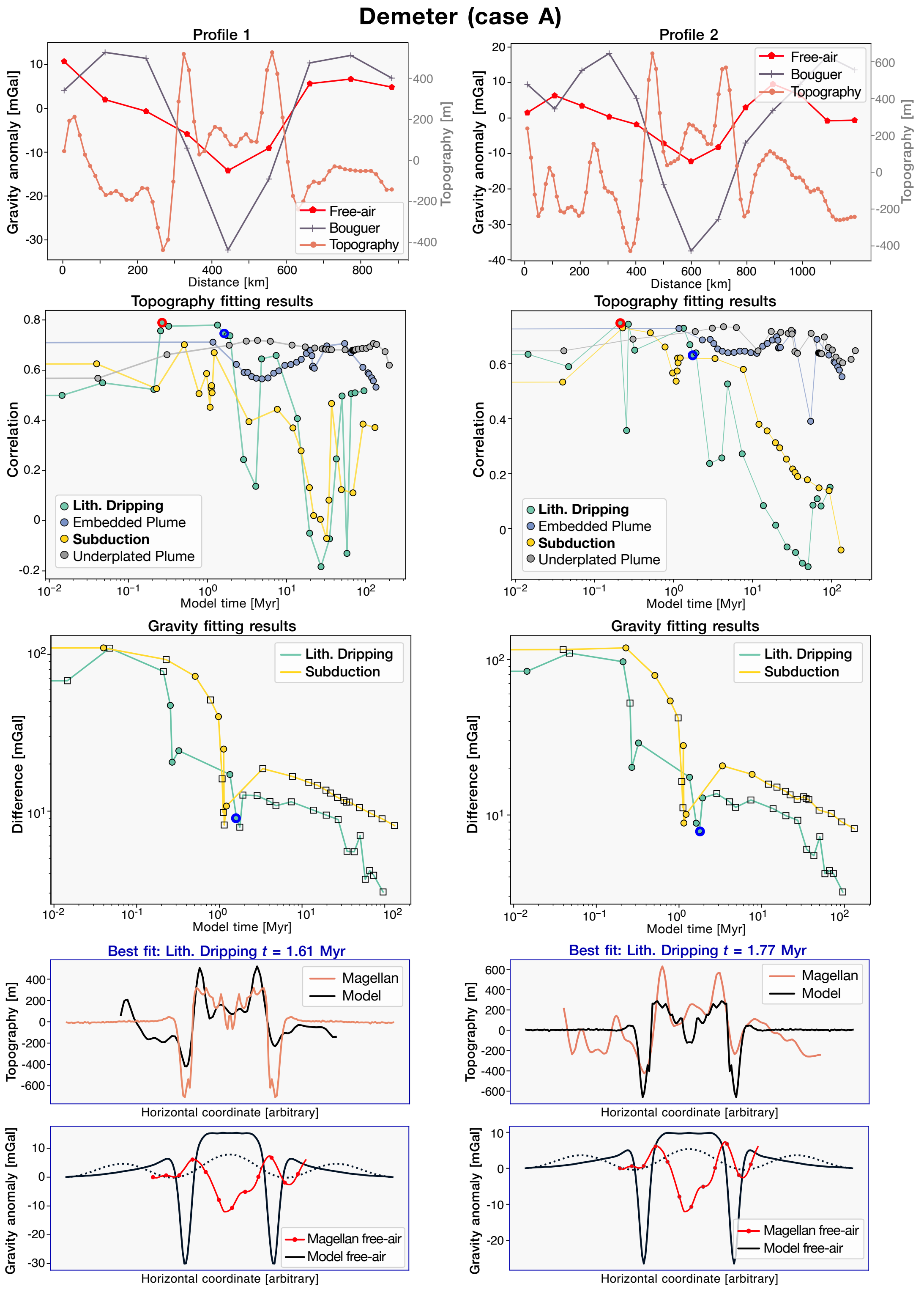

Figure S11: **Topography and gravity fitting results for Demeter corona** (295.2**E**, 54.2 **N**)**.** The figure description is the same as in figure S5.

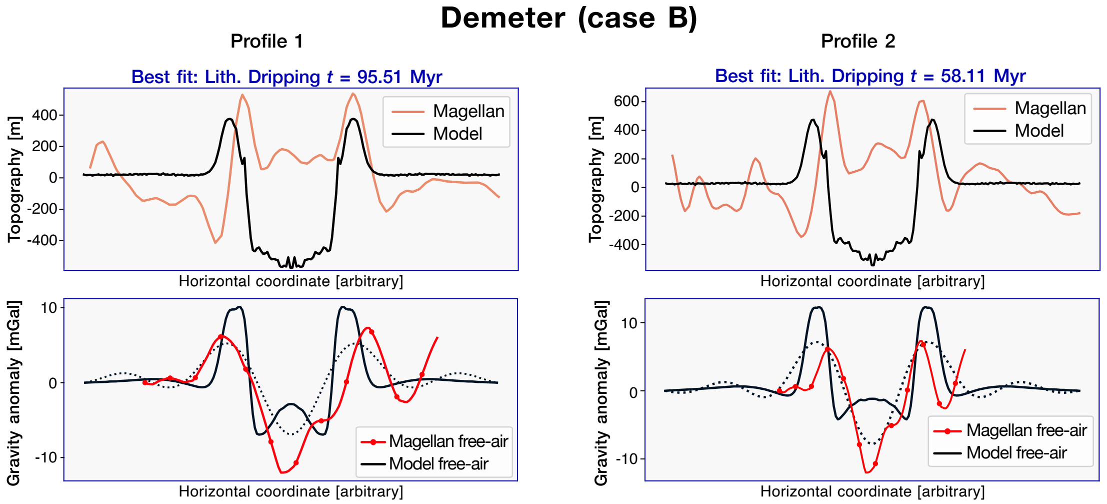

Figure S12: **Alternative topography and gravity fitting results for Demeter corona** (295.2**E,** 54.2 **N).** These are alternative solutions for Demeter obtained when searching for the best fitting gravity signal without constraints on the quality of the topography fit, to be compared to the results in figure S11. The description of the two rows is the same as that for the bottom rows in figure S6.

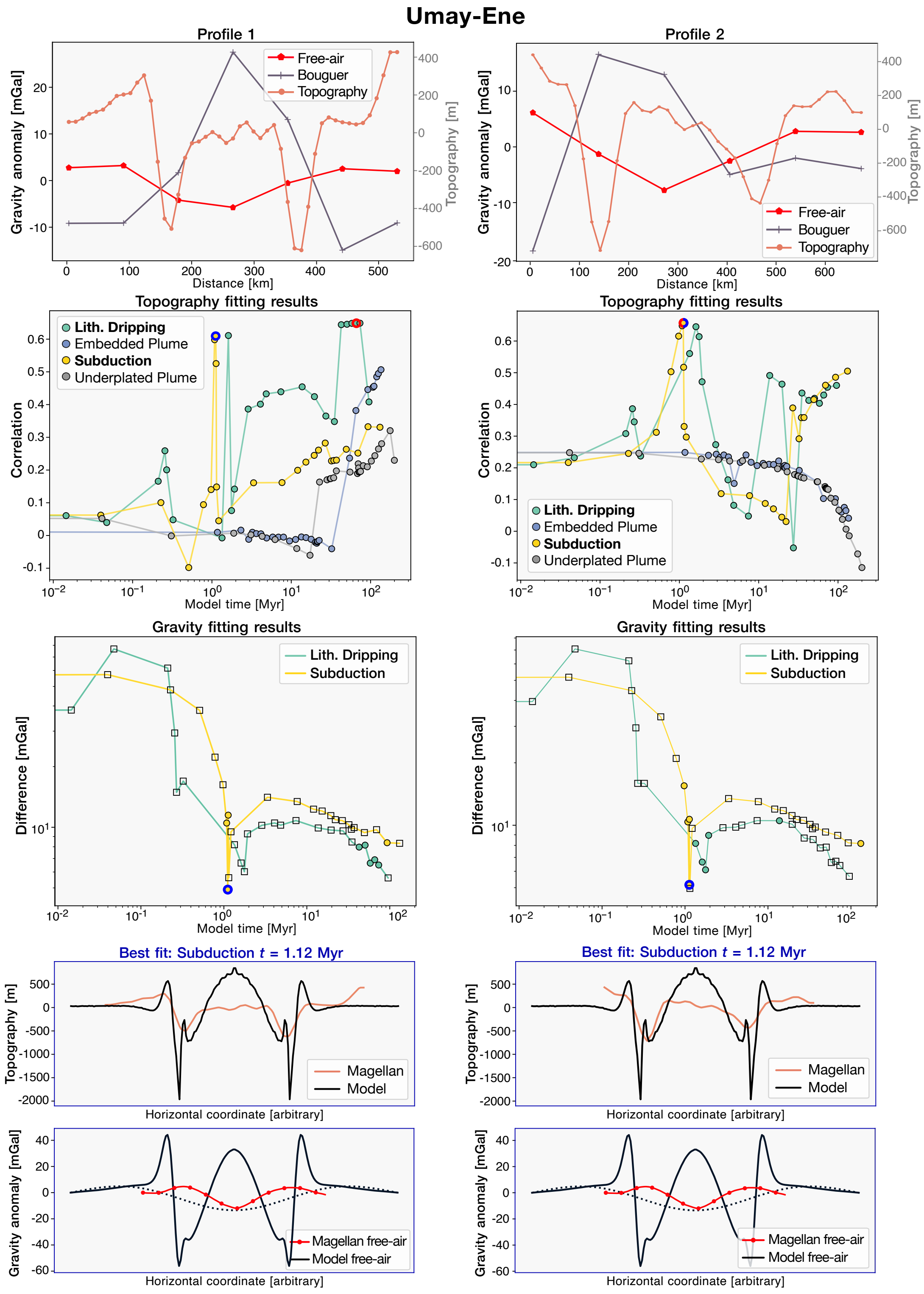

Figure S13: **Topography and gravity fitting results for Umay-Ene corona** (50.6**E**, 27.7**S**). The first profile (left column) is also shown in Figure 6D in the main paper. The further figure description is the same as in figure S8.

Supplementary Data

The Supplementary Data is available for download on Zenodo (https://doi.org/10.5281/zenodo.13357274.

Data S1. 3D Gridded density data of geodynamic models

This dataset includes `.mat` files (MATLAB data format) for several snapshots across all four geodynamic models explored in this study. The folders separate the different geodynamic regimes. The `.mat` files in each folder contain the following information: domain dimensions (`xsize`, `ysize`, and `zsize` in meters, with y representing depth), domain geometry ($x \cdot y \cdot z$ grid), time in units of million years (`ntime`), the 3D density grid (`rho3D`), and the 2D surface density grid (`rhosurf`). The 3D gridded data were used to compute surface gravity anomalies, as described in the Methods section of the main paper. The dataset also includes the file `models_timesteps.json`, which contains arrays of all available timesteps and the corresponding model times for each geodynamic model.

Data S2. Table with corona classification and gravity resolutions

This `.csv` file contains the database accompanying this study. It contains two sheets: the main sheet closely resembles Extended Data Table 1 as given in the paper, and describes 75 corona features considered resolved in the Magellan gravity data (see Methods in main paper). The following information is provided for these coronae: central coordinates, diameter (based on fracture annuli, from (*5*)), topographic group (from (*5*)), Magellan gravity resolution at central coordinate, Magellan free air gravity anomaly (global value and local anomaly relative to immediate surroundings), Bouguer gravity anomaly (global value and local anomaly relative to immediate surroundings), classification and tectonic processes inferred in this study, and corona activity classifications from other studies (*14, 15, 23*), if applicable. The second sheet contains all 740 corona features from (*5*), their central coordinates, diameters, the Magellan gravity resolution at central coordinate, and the predicted resolution of the VERITAS gravity data (*24*).

The process of computing the global gravity values and local anomaly relative to immediate surroundings is given in the Methods of the manuscript. The classification codes relate to the topography and gravity of each corona, they are defined as follows:

Table S1: **Table with corona classification in the Magellan database.** 75 coronae, diameter D based on fracture annuli (*5*), coordinates, Magellan free air gravity anomaly (global value and local anomaly relative to immediate surroundings), Bouguer gravity anomaly (global value and local anomaly relative to immediate surroundings), classification and tectonic processes inferred (Ative CR: 'Active plume-induced crustal recycling' and Active UE: 'Active underplated plume/embedded'), if applicable. For the comparison to the gravity field resolution, the "topographic radius" is used ($R_{\text{topo}} = 1.2 \cdot R_{\text{fracture}}$, see Methods). The classification codes are defined as follows: 1) Chasmata-related corona with a (partial) topographic trench and a local free air gravity anomaly (*active*; N=16); 2) Isolated corona with a (partial) topographic trench and a local free air gravity anomaly (*active*; N=18); 3) Isolated corona with a raised rim and elevated interior and a local free air gravity anomaly (*active*; N=12); 4) Isolated corona with a (partial) topographic trench and no or a negative free air anomaly (N=8). These four groups are compared with geodynamic modeling data in our paper. Additional classes are: 5) Isolated coronae with other topographic signatures (rim-only or rim with inner dome) and a positive free air anomaly (*active*; N=6); 6) Isolated corona with no (partial) topographic trench and no or a negative free air anomaly (N=15). In the final column, the term '(p)' at coronae with inferred plume-induced crustal recycling stands for the trench covering less than the full corona circumference (i.e., partial). Coronae marked with * are those with strong local gravity anomalies, but where the calculated value (algorithm described in Methods) is obscured by nearby geological features like a volcanic Mons or chasmata (see Methods). In contrast, ** indicates features where our algorithm detects anomalies, but regional analysis refutes this due to the presence of local geological features with strong gravity anomalies. A supplementary table in CSV format available on Zenodo (https://doi.org/10.5281/zenodo.13357274) includes additional information such as the topographic group classification according to (*5*), the topographic radius, the resolution of the Magellan gravity data, the expected resolution of the VERITAS gravity data, and, where applicable, corona activity classifications from other studies (*14, 15, 23*), as well as corona elastic thickness estimates from (*2*).

				Free air $\triangle g$ [mGal]		Bouguer $\triangle g$ [mGal]			
**Name**	**Lat**	**Long**	D **[km]**	**Global**	**Local**	**Global**	**Local**	**Class**	**Inferred tectonics**
Artemis	-32.5	132.1	2500	70.6	73.9	-27.0	-47.2	2	Active CR
Heng-O	1.8	354.6	1070	28.0	35.7	2.4	1.7	2	Active CR
Thermuthis (outer) **	-8.9	33	970	40.5	50.5**	4.4	4.7**	6	
Atahensik (Latona)	-19.7	170.6	920	83.6	60.6	-33.8	-34.9	1	Active CR
Quetzalpetlatl	-68.2	356.2	850	88.2	59.5	-41.2	-50.8	2	Active CR
48S75W	-47.5	285.4	690	24.5	15.2	-11.9	-20.9	5	
Dunne-Musun	-60.1	85	680	30.8	22.1	-20.1	-21.8	2	Active CR
Ceres*	-16.1	151.2	670	-4.6	-28.4*	7.7	-2.2*	1	Active CR
Poloznitza	-0.1	301.7	670	-14.5	-28.1	3.6	-6.3	6	
7N7E	6.6	6.5	660	-38.8	-28.6	149.5	61.9	6	
50S96W	-49.7	264.3	650	-23.0	-9.4	3.3	1.9	6	
Copia	-42	74.3	630	-2.5	14.0	-20.0	-25.2	2	Active CR (p)
Zisa	12	221	620	81.8	74.6	-54.0	-58.9	1	Active CR (p)
Ekhe-Burkhan	-50	40	610	48.7	30.8	-8.5	-10.2	3	Active UE
Ambar-ona	-70	82	610	-9.0	-15.7	8.4	6.4	4	
70N87W	69.6	273.4	610	11.6	16.4	20.9	11.7	5	
Zemina*	-12	185.9	590	14.1	-19.9*	8.7	4.5*	1	Active CR (p)
Nabuzana**	-8.6	47.4	580	-1.8	15.1**	3.5	4.5**	4	
Atete	-15.5	243.9	580	79.4	74.1	-10.1	-10.6	1	Active CR (p)
30N165E	29.6	165.2	560	-37.3	-15.2	15.6	12.3	6	
Calakomana**	6.5	43.8	555	34.2	22.2**	25.8	20.0**	4	
Bachue	73.3	259.7	550	67.5	66.3	-69.5	-76.9	2	Active CR (p)
Lilwani	-29.7	272	550	-17.3	-14.8	-10.9	-8.2	1	Active CR (p)
10N14W	9.7	345.7	550	-42.0	-13.7	8.1	9.0	6	

Table continued on next page ...

Table continued from previous page ...									
				**Free air △g [mGal]**		**Bouguer △g [mGal]**			
**Name**	**Lat**	**Long**	D [km]	**Global**	**Local**	**Global**	**Local**	**Class**	**Inferred tectonics**
10N14W	9.7	345.7	550	-42.0	-13.7	8.1	9.0	6	
Beiwe	52.8	307.3	550	-8.6	16.9	-25	-23.2	3	Active UE
Demeter	54.2	295.2	540	-51.3	-18.5	-21.5	-18.4	4	
24S83W	-23.6	277.3	525	-27.2	-31.2	10.4	4.0	6	
Eithinoha	-57	8	520	45.2	20.9	-27.4	-19.2	1	Active CR (p)
Nightingale	63.4	130.5	520	-1.4	-26.5*	-50.0	-57.2	2	Active CR (p)
Maram	-7.7	221.7	520	118.5	112.2	-50.8	-50.4	1	Active CR (p)
Taranga	16.4	252	520	114.6	126.8	-1.1	-2.4	1	Active CR
Shiwanokia	-42	279.5	520	89.4	73.5	-80.1	-88.6	3	Active UE
Lalohonua	-24.1	250.6	510	56.5	56.7	-45.8	-49.8	3	Active UE
Pavlova	14.6	40	500	64.6	38.1	-52.9	-57.5	3	Active UE
Mukylchin	-12.4	46	500	-37.2	-12.0	36.2	35.6	6	
Rananeida	62.9	264.4	500	-35.0	-26.4	14.6	13.2	6	
Otygen	-57.2	31.1	490	30.5	13.0	-21.0	-26.2	2	Active CR
Isong	11.3	49	490	75.2	67.1	-25.9	-31.5	3	Active UE
Marzyana	-53	67.5	490	52.7	25.2	-16.4	-13.7	1	Active CR
Kamadhenu	20.9	136.3	490	-14.9	-7.6	5.1	3.4	5	
43S33W	-43.2	326.9	490	54.5	48.0	-33.7	-46.5	2	Active CR (p)
Ninmah	16.6	48.6	480	49.9	40.2	-41.0	-41.5	3	Active UE
Vacuna	60.4	95.5	480	26.7	10.2*	1.3	-6.4*	5	
Bau	52.9	258.7	480	0.3	13.8	-6.0	-10.8	2	Active CR (p)
Iweridd	-20.5	309.2	480	99.5	78.7	-43.1	-45.8	1	Active CR
Javine	-5	251	475	44.5	51.2	-21.8	-16.9	2	Active CR (p)
Fatua	-16.3	17.9	470	33.1	23.8	-21.3	-20.3	2	Active CR
Bibi-Patma	-47	302	465	30.5	23.9	-28.0	-25.2	3	Active UE
Gaia*	3.9	21.6	460	10.4	16.0	8.3	8.1*	2	Active CR
Ma	-22.5	56.8	460	-15.8	7.8*	-16.1	-16.5	2	Active CR
2S6E	-2.3	5.8	450	-38.1	-21.5	-6.5	-8.5	6	
Cybele**	-7.6	21	450	11.5	11.5**	12.8	15.8**	4	
12N45E	12	44.6	450	-8.2	-30.1	31.3	37.2	6	
Lengdin	2.5	223	450	58.2	54.6	-28.3	-31.5	1	Active CR (p)
Miralaidji	-13.9	163.8	440	-21.5	-35.1*	-27.0	-28.6*	1	Active CR (p)
Feronia	68.4	279.7	430	9.1	20.2	-9.9	-11.4	2	Active CR (p)
Omosi-Mama	64.3	306.7	430	-39.8	-34.2	77.2	15.9	6	
Selu	-42.2	6.4	425	34.4	23.2	-40.8	-37.0	3	Active UE
Anahit	77.3	278	425	-25.4	-26.9	-3.9	-11.0	4	
Silvia	12.7	355.7	420	-15.0	-26.9	-14.6	-24.3	5	
Eve	-32.1	359.4	420	43.8	32.2	-60.6	-95.8	2	Active CR (p)
Semiramus	-36.9	292.7	410	56.4	49.1	-16.8	-18.9	1	Active CR (p)
Aruru	9.3	262.3	405	37.0	21.9	-55.3	-61.2	3	Active UE
Haumea	54	21.8	400	18.7	-7.6	49.1	27.1	4	
Ereshkigal	21.2	84.1	400	13.4	24.6	-40.8	-49.6	3	Active UE
Perchta	16.8	234.3	400	102.6	98.8	-26.0	-22.1	1	Active CR
Kaltash	0.5	75	390	-19.5	-25.6	30.3	26.2	6	
Umay-ene	-27.7	50.6	380	-19.2	-26.0	6.5	8.9	4	
37N60E	37.4	59.6	380	15.2	20.7	5.2	-10.8	5	
4N79E	4.2	78.7	380	-22.1	-19.5	44.7	6.1	4	
Pani	19.7	231.8	380	65.8	55.5	-13.7	-15.7	1	Active CR (p)
Nefertiti	36.4	47.9	365	68.1	47.1	-82.0	-92.4	2	Active CR (p)
Didilia	18.6	37.5	360	67.1	39.0	-40.1	-36.9	3	Active UE
Libera	12.8	24.2	350	-8.1	-27.3	-5.4	3.2	6	
Branwen	27	35	350	26.6	20.1	19.9	15.7	2	Active CR

1. Chasmata-related corona with a (partial) topographic trench and a local free air gravity anomaly (”active”)
2. Isolated corona with a (partial) topographic trench and a local free air gravity anomaly (”active”)
3. Isolated corona with a raised rim and elevated interior and a local free air gravity anomaly (”active”)
4. Isolated corona with a (partial) topographic trench and no or a negative free air anomaly
5. Isolated coronae with other topographic signatures (rim-only or rim with inner dome) and a positive free air anomaly (”active”)
6. Isolated corona with no (partial) topographic trench and no or a negative free air anomaly

In the *inferred tectonic scenario* column, the term ‘(p)’ mean that the topographic trench, and therefore inferred plume-induced crustal recycling scenario, only covers part of the asymmetric corona circumference. Coronae marked with * denote those with strong local high gravity anomalies, but where the calculated value (algorithm described in Methods) is obscured by nearby geological features like a volcanic Mons or chasmata (see Methods), detecting only a small, no, or a negative anomaly. In contrast, ** indicates features where our algorithm detects anomalies, but regional analysis refutes this due to the presence of local geological features with strong gravity anomalies.

The three studies on corona activity included in this dataset (*14, 15, 23*) employed different approaches and terminology for investigating ongoing dynamics at Venus coronae. A key distinction among these studies is their reliance on outdated and incomplete corona datasets [e.g., (*6,61*)], while we use a recently developed corona database (*5*). This database, based on a systematic approach to identifying corona features, includes 740 recorded features with updated information such as central coordinates, dimensions, and topographic classifications compared to previous catalogs [for more details see (*5*)].

(*23*) used the Type 1 database (408 features) from (*6*) to assess peak free-air gravity values within coronae and visually identified the presence or absence of local anomalies. Any corona with a visual anomaly, positive or negative, was labeled “uncompensated” (124 counts), while 19 were considered clearly “compensated” (inactive). The remaining 264 coronae were unclassified. Our activity classification generally aligns with the “uncompensated” category. However, we note the

inclusion of coronae with negative gravity anomalies in the "uncompensated" category in (*23*), which differs from our approach of targeting positive free-air gravity anomalies as these likely relate to plume activity. We also observe discrepancies where some "compensated" coronae are linked to detected free-air gravity anomalies by our algorithm, and vice versa (see Data S2).

(*14*) examined topography and gravity of Type 1 and Type 2 coronae [from (*6*)] within the Beta-Atla-Themis region, filtering the datasets between harmonic degrees 15 and 44 (i.e., large wavelengths only). They identified seven coronae as "active," characterized by elevated topography and free-air gravity anomalies, and negative Bouguer anomalies. While these seven align with our 'activity' classification, we identify additional active coronae in the BAT region and propose specific active tectonic processes at work.

(*15*) analyzed the 133 largest named coronae from the USGS nomenclature (*61*) based solely on visual inspection of surface topography. They suggested "active" plume-lithosphere interactions for coronae with (partial) trenches. Many coronae with a raised rim and elevated interior (group 3 in our active classification) were often considered unclassified, while here, we quantitatively demonstrate how incorporating gravity data supports the presence of active plumes beneath these topographic features. For example, Pavlova (fig. S9), Beiwe (307.3°E, 52.8°N), and Shiwanokia (179.5°E, 42°S), were labeled 'unclassified' in (*15*), but are here shown to match both the topography and gravity signature of an active embedded/underplated plume. Aruru (262.3°E, 9.3°N) and Ereshkigal (84.1°E, 21.2°N), also classified as active embedded/underplated plume scenarios (table S1), were termed 'inactive' in (*15*) due to their topographic profile classification (rim-only) versus that used in our work (rim surrounding a raised interior compared to the surroundings), informed by the recent coronae database (*5*). Another discrepancy is that (*15*) often refrained from classifying coronae embedded within major rift axes (chasmata), such as Maram (221.7°E, 7.7°S) and Taranga (252°E, 16.4°N). In line with the recent topographic classification by (*5*), we include chasmata in topographic profiles if they encircle a corona, and make a clear distinction between rift-related and isolated coronae for which 'active plume-induced crustal recycling is proposed.

In summary, our study advances previous research by using an updated corona dataset, a systematic and quantitative analysis of topography and gravity combined, and alignment with geodynamic model predictions to propose varied styles of active plume-lithosphere tectonic processes at coronae with clear evidence of active buoyant material underneath.

Data S3. 2D Gridded gravity and topography of geodynamic models.

This dataset includes netCDF4 (.nc) files for several snapshots across four geodynamic models explored in this study. Each file contains 2D regular grids for several different time snapshots of either free-air gravity anomaly, Bouguer gravity anomaly, or topography.
File naming is as follows: `xTYPE_MODEL.nc`. `TYPE` can be: `grav` for free-air gravity anomaly, `bouguer` for Bouguer gravity anomaly, `topo` for topography. `MODEL` indicates one of the four geodynamic end-member scenarios: `Embedded_Plume`, `Lith_Dripping`, `Subduction`, `Underplated_Plume`.
Each file contains the corresponding quntity specified in `TYPE` for several time steps. As such each netCDF file contains data represented by three variables:

1. time: the time of the snapshots, in years. This has variable size among models.
2. x: the x-coordinate, in meters. Size = 300 indexes for gravity and 405 for topography.
3. y: the y-coordinate, in meters. Size = 300 indexes for gravity and 405 for topography.

We also provide a simple Python script demonstrating how to read and manipulate the data using the `netCDF4` library or the `xarray` library: `test_read.py`

REFERENCES AND NOTES

1. T. Rolf, M. Weller, A. Gülcher, P. Byrne, J. G. O'Rourke, R. Herrick, E. Bjonnes, A. Davaille, R. Ghail, C. Gillmann, A.-C. Plesa, S. Smrekar, Dynamics and evolution of Venus' mantle through time. *Space Sci. Rev.* **218**, 70 (2022).

2. S. E. Smrekar, C. Ostberg, J. G. O'Rourke, Earth-like lithospheric thickness and heat flow on Venus consistent with active rifting. *Nat. Geosci.* **16**, 13–18 (2023).

3. R. R. Herrick, S. Hensley, Surface changes observed on a Venusian volcano during the Magellan mission. *Science* **379**, 1205–1208 (2023).

4. D. Sulcanese, G. Mitri, M. Mastrogiuseppe, Evidence of ongoing volcanic activity on Venus revealed by Magellan radar. *Nat. Astron.* **8**, 973–982 (2024).

5. A. J. P. Gülcher, L. Sabbeth, E. Stofan, S. E. Smrekar, Coronae on Venus: An updated global database and insights into morphology, spatial distribution, geological setting, and lithospheric properties. *J. Geophys. Res. Planets,* 101029/2024J/JE008749 (2025).

6. E. R. Stofan, S. E. Smrekar, S. W. Tapper, J. E. Guest, P. M. Grindrod, Preliminary analysis of an expanded corona database for Venus. *Geophys. Res. Lett.* **28**, 4267–4270 (2001).

7. L. S. Glaze, E. R. Stofan, S. E. Smrekar, S. M. Baloga, Insights into corona formation through statistical analyses: Insights into corona formation. *J. Geophys. Res. Planets.* **107**, 18-1–18-12 (2002).

8. E. R. Stofan, D. L. Bindschadler, J. W. Head, E. M. Parmentier, Corona structures on Venus: Models of origin. *J. Geophys. Res.* **96**, 20933, 20946 (1991).

9. D. M. Janes, S. W. Squyres, D. L. Bindschadler, G. Baer, G. Schubert, V. L. Sharpton, E. R. Stofan, Geophysical models for the formation and evolution of coronae on Venus. *J. Geophys. Res.* **97**, 16055 (1992), 16067.

10. S. W. Squyres, D. L. Bindschadler, D. M. Janes, G. Schubert, V. L. Sharpton, E. R. Stofan, Morphology and evolution of coronae and ovoids on Venus. in *Abstracts of papers submitted to the Twenty-second lunar and planetary science conference* (1992), vol. 22, 1307–1308.

11. D. M. Janes, S. W. Squyres, Viscoelastic relaxation of topographic highs on Venus to produce coronae. *J. Geophys. Res. Planets.* **100**, 21173–21187 (1995).

12. D. M. Koch, M. Manga, Neutrally buoyant diapirs: A model for venus coronae. *Geophys. Res. Lett.* **23**, 225–228 (1996).

13. A. Davaille, S. E. Smrekar, S. Tomlinson, Experimental and observational evidence for plume-induced subduction on Venus. *Nat. Geosci.* **10**, 349–355 (2017).

14. A. J. Dombard, C. L. Johnson, M. A. Richards, S. C. Solomon, A magmatic loading model for coronae on Venus. *J. Geophys. Res. Planets.* **112**, doi.org/10.1029/2006JE002731 (2007).

15. A. J. P. Gülcher, T. V. Gerya, L. G. J. Montési, J. Munch, Corona structures driven by plume-lithosphere interactions and evidence for ongoing plume activity on Venus. *Nat. Geosci.* **13**, 547–554 (2020).

16. J. Schools, S. Smrekar, Formation of coronae topography and fractures via plume buoyancy and melting. *Earth Planet. Sci. Lett.* **633**, 118643 (2024).

17. T. Hoogenboom, G. A. Houseman, Rayleigh-Taylor instability as a mechanism for corona formation on Venus. *Icarus* **180**, 292–307 (2006).

18. D. Piskorz, L. T. Elkins-Tanton, S. E. Smrekar, Coronae formation on Venus via extension and lithospheric instability. *J. Geophys. Res. Planets* **119**, 2568–2582 (2014).

19. S. E. Smrekar, E. R. Stofan, Corona formation and heat loss on venus by coupled upwelling and delamination. *Science* **277**, 1289–1294 (1997).

20. T. V. Gerya, Plume-induced crustal convection: 3D thermomechanical model and implications for the origin of novae and coronae on Venus. *Earth Planet. Sci. Lett.* **391**, 183–192 (2014).

21. A. J. P. Gülcher, T.-Y. Yu, T. V. Gerya, Tectono-magmatic evolution of asymmetric coronae on venus: topographic classification and 3D thermo-mechanical modeling. *J. Geophys. Res. Planets* **128**, e2023JE007978 (2023).

22. G. Schubert, D. T. Sandwell, A global survey of possible subduction sites on Venus. *Icarus* **117**, 173–196 (1995).

23. C. L. Johnson, M. A. Richards, A conceptual model for the relationship between coronae and large-scale mantle dynamics on Venus. *J. Geophys. Res.* **108**, 5058 (2003).

24. F. Giuliani, D. Durante, G. Cascioli, F. De Marchi, L. Iess, E. Mazarico, S. Smrekar, Mapping Venus's gravity field with the VERITAS mission. *Planet. Sci. J.* **6**, 13 (2025).

25. A. S. Konopliv, W. B. Banerdt, W. L. Sjogren, Venus Gravity: 180th degree and order model. *Icarus* **139**, 3–18 (1999).

26. M. A. Wieczorek, Gravity and Topography of the Terrestrial Planets. *Tr. Geophys.* **10**, 153–193 (2015).

27. D. T. Sandwell, G. Schubert, Evidence for retrograde lithospheric subduction on Venus. *Science* **257**, 766–770 (1992).

28. C. L. Johnson, D. T. Sandwell, Lithospheric flexure on Venus. *Geophys. J. Int.* **119**, 627–647 (1994).

29. S. E. Smrekar, E. R. Stofan, Origin of corona-dominated topographic rises on Venus. *Icarus* **139**, 100–115 (1999).

30. P. J. McGovern, M. E. Rumpf, J. R. Zimbelman, The influence of lithospheric flexure on magma ascent at large volcanoes on Venus. *J. Geophys. Res. Planets* **118**, 2423–2437 (2013).

31. S. Smrekar, S. Hensley, R. Nybakken, M. S. Wallace, D. Perkovic-Martin, T.-H. You, D. Nunes, J. Brophy, T. Ely, E. Burt, M. D. Dyar, J. Helbert, B. Miller, J. Hartley, P. Kallemeyn, J. Whitten, L. Iess, M. Mastrogiuseppe, M. Younis, P. Prats, M. Rodriguez, E. Mazarico, VERITAS (Venus Emissivity, Radio Science, InSAR, Topography, and Spectroscopy): A Discovery Mission, in *2022 IEEE Aerospace Conference (AERO)* (IEEE; 2022), pp. 1–20; 10.1109/AERO53065.2022.9843269.

32. G. Cascioli, S. Hensley, F. D. Marchi, D. Breuer, D. Durante, P. Racioppa, L. Iess, E. Mazarico, S. E. Smrekar, The determination of the rotational state and interior structure of Venus with VERITAS. *Planet. Sci. J.* **2**, 220 (2021).

33. G. Cascioli, J. P. Renaud, E. Mazarico, D. Durante, L. Iess, S. Goossens, S. Smrekar, Constraining the Venus interior structure with future VERITAS measurements of the gravitational atmospheric loading. *Planet. Sci. J.* **4**, 65 (2023).

34. P. Rosenblatt, C. Dumoulin, J.-C. Marty, A. Genova, Determination of Venus' interior structure with EnVision. *Remote Sens.* **13**, 1624 (2021).

35. E. Mazarico, L. Iess, G. Cascioli, D. Breuer, S. Hensley, S. E. Smrekar, The VERITAS Gravity Science Investigation, In *AGU Fall Meeting Abstracts* (2021), vol. 2021, pp. P35H-2218; https://agu.confex.com/agu/fm21/meetingapp.cgi/Paper/858214.

36. T. V. Gerya, D. A. Yuen, Robust characteristics method for modelling multiphase visco-elasto-plastic thermo-mechanical problems. *Phys. Earth Planet. In.* **163**, 83–105 (2007).

37. T. V. Gerya, R. J. Stern, M. Baes, S. V. Sobolev, S. A. Whattam, Plate tectonics on the Earth triggered by plume-induced subduction initiation. *Nature* **527**, 221–225 (2015).

38. T. V. Gerya, Dynamical instability produces transform faults at mid-ocean ridges. *Science* **329**, 1047–1050 (2010).

39. D. L. Turcotte, G. Schubert, *Geodynamics* (Cambridge Univ. Press,2012).

40. F. S. Anderson, S. E. Smrekar, Global mapping of crustal and lithospheric thickness on Venus. *J. Geophys. Res. E: Planets* **111**, doi.org/10.1029/2004JE002395 (2006).

41. F. D. Esteban, L. Li, V. C. Oliveira Jr, A. Pesce, N. Shea, S. R. Soler, M. Tankersley, L. Uieda, Harmonica v0.6.0: Forward modeling, inversion, and processing gravity and magnetic data (Zenodo, 2023); https://doi.org/10.5281/zenodo.7690145 .

42. E. Mazarico, A. Genova, S. Goossens, F. G. Lemoine, G. A. Neumann, M. T. Zuber, D. E. Smith, S. C. Solomon, The gravity field, orientation, and ephemeris of Mercury from MESSENGER observations after three years in orbit. *J. Geophys. Res. Planets* **119**, 2417–2436 (2014).

43. S. Goossens, T. J. Sabaka, M. A. Wieczorek, G. A. Neumann, E. Mazarico, F. G. Lemoine, J. B. Nicholas, D. E. Smith, M. T. Zuber, High-resolution gravity field models from GRAIL data and implications for models of the density structure of the moon's crust. *J. Geophys. Res. Planets* **125**, e2019JE006086 (2020).

44. F. G. Lemoine, S. Goossens, T. J. Sabaka, J. B. Nicholas, E. Mazarico, D. D. Rowlands, B. D. Loomis, D. S. Chinn, G. A. Neumann, D. E. Smith, M. T. Zuber, GRGM900C: A degree 900 lunar gravity model from GRAIL primary and extended mission data. *Geophys. Res. Lett.* **41**, 3382–3389 (2014).

45. A. Konopliv, R. Park, A. Ermakov, The Mercury gravity field, orientation, love number, and ephemeris from the MESSENGER radiometric tracking data. *Icarus* **335**, 113386 (2020).

46. S. Buoninfante, M. Milano, B. Negri, C. Plainaki, G. Sindoni, M. Fedi, Gravity evidence for a heterogeneous crust of Mercury. *Sci. Rep.* **13**, 19854 (2023).

47. L. Sabbeth, M. Carrington, S. Smrekar, Constraints on corona formation from an analysis of topographic rims and fracture annuli. *Earth Planet. Sci. Lett.* **633**, 118568 (2024).

48. R. R. Herrick, D. L. Stahlke, V. L. Sharpton, Fine-scale Venusian topography from Magellan stereo data. *Eos, Trans. AGU* **93**, 125–126 (2012).

49. P. Virtanen, R. Gommers, T. E. Oliphant, M. Haberland, T. Reddy, D. Cournapeau, E. Burovski, P. Peterson, W. Weckesser, J. Bright, S. J. van der Walt, M. Brett, J. Wilson, K. J. Millman, N. Mayorov, A. R. J. Nelson, E. Jones, R. Kern, E. Larson, C. J. Carey, İ. Polat, Y. Feng, E. W. Moore, J. VanderPlas, D. Laxalde, J. Perktold, R. Cimrman, I. Henriksen, E. A. Quintero, C. R. Harris, A. M. Archibald, A. H. Ribeiro, F. Pedregosa, P. van Mulbregt, SciPy 1.0 Contributors, SciPy 1.0: Fundamental Algorithms for Scientific Computing in Python. *Nat. Methods* **17**, 261–272 (2020).

50. M. J. D. Powell, An efficient method for finding the minimum of a function of several variables without calculating derivatives. *Comput. J.* **7**, 155–162 (1964).

51. Itseez, Open Source Computer Vision Library (2015); https://github.com/itseez/opencv.

52. E. R. Stofan, V. L. Sharpton, G. Schubert, G. Baer, L. Bindschadler, D. M. Janes, W. Squyres, Global distribution and Characteristics of coronae and related features on Venus: implications for origin and relation to mantle processes. *J. Geophys. Res.* **97**, 347–378 (1992).

53. S. W. Asmar, J. W. Armstrong, L. Iess, P. Tortora, Spacecraft Doppler tracking: Noise budget and accuracy achievable in precision radio science observations. *Radio Sci.* **40**, n/a–n/a (2005).

54. L. Iess, M. Di Benedetto, N. James, M. Mercolino, L. Simone, P. Tortora, Astra: Interdisciplinary study on enhancement of the end-to-end accuracy for spacecraft tracking techniques. *Acta Astronaut.* **94**, 699–707 (2014).

55. P. Cappuccio, V. Notaro, A. di. Ruscio, L. Iess, A. Genova, D. Durante, I. di. Stefano, S. W. Asmar, S. Ciarcia, L. Simone, Report on First Inflight Data of BepiColombo's Mercury Orbiter Radio Science Experiment. *IEEE Trans. Aerosp. Electron. Syst.* **56**, 4984–4988 (2020).

56. G. Cascioli, D. Durante, E. Mazarico, M. Wallace, S. Hensley, S. Smrekar, Improving the VERITAS orbit reconstruction using radar tie points. *J. Spacecr. Rockets* **60**, 366–373 (2023).

57. P. G. Ford, G. H. Pettengill, Venus topography and kilometer-scale slopes. *J. Geophys. Res. Planets* **97**, 13103–13114 (1992).

58. A. J. P. Gülcher, Coronae on Venus: An updated global database and insights into morphology, spatial distribution, geological setting, and lithospheric properties, (Zenodo; 2025); doi:10.5281/zenodo.14793392.

59. A. J. P. Gülcher, Venus coronae topographic (a)symmetry classification (from Gülcher et al., 2023, JGR Planets) (Zenodo, 2023); doi:10.5281/zenodo.8316805.

60. G. H. Pettengill, P. G. Ford, W. T. K. Johnson, R. K. Raney, L. A. Soderblom, Magellan: Radar performance and data products. *Science* **252**, 260–265 (1991).

61. U.S. Geological Survey, Gazetteer of Planetary Nomenclature (2023); https://planetarynames.wr.usgs.gov/Page/VENUS/target.
